# In Situ Electrically Resettable Field‐Effect Transistor Biosensors for Continuous and Multiplexed Neurotransmitter Detection

**DOI:** 10.1002/advs.202504497

**Published:** 2025-05-28

**Authors:** Bo Xiao, Tingxian Li, Xianmao Cao, Yang Zhang, Jianping He, Mengmeng Xiao, Zhiyong Zhang

**Affiliations:** ^1^ Hunan Institute of Advanced Sensing and Information Technology Xiangtan University Hunan 411105 China; ^2^ Key Laboratory for the Physics and Chemistry of Nanodevices and Center for Carbon‐based Electronics Department of Electronics Peking University Beijing 100871 China; ^3^ School of Integrated Circuits Beijing University of Posts and Telecommunications Beijing 100876 China

**Keywords:** allosteric DNA, field‐effect transistor, neurotransmitters, regenerable biosensors

## Abstract

Accurate, real‐time detection of neurotransmitters is crucial for elucidating the mechanisms of brain function and tracking the progression of neurological diseases. Biosensors usually face poor reusability induced by difficulties in probe‐target separation, hindering their application for continuous monitoring. In this work, a semiconducting carbon nanotube (CNT) film field‐effect transistor (FET) biosensor is developed using pH‐sensitive aptamers as probes to capture targets. Through tuning the potential of on‐chip palladium electrodes, the biosensor facilitates in situ pH modulation and recovery of the sensor interface, which enables an electrically resettable biosensor. The fabricated sensor demonstrates exceptional sensitivity (with femtomolar‐level detection limits), high selectivity (specific responses are 20 times stronger than non‐specific ones), and excellent reusability (over ten reuse cycles). Furthermore, in vitro detection demonstrates that the biosensor arrays, incorporating regional modifications and pH‐sensitive probes, enable the simultaneous detection of several neurotransmitters, including dopamine, serotonin, histamine, and glutamate in complex biological samples. The combination of microfluidic techniques further lowers non‐specific adsorption and cross‐reactivity, ensuring reliable, repeatable real‐time detection. The resettable CNT FET biosensor array holds significant promise for advancing the monitoring of neurotransmitter dynamics, serving as a powerful tool for the early diagnosis and management of neurological disorders.

## Graphical abstract

The research team integrated a potentiometrically controlled pH electrode with carbon nanotube (CNT) field‐effect transistor (FET) biosensors on a single chip using compatible processes. By employing zonal modification and optimized pH‐sensitive probes, they achieved simultaneous quadruple‐target detection and tenfold reusability under physiological conditions, laying the foundation for next‐generation real‐time diagnostic tools.

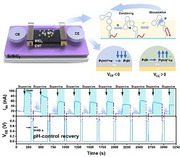

## 1 Introduction

**Figure 1:**
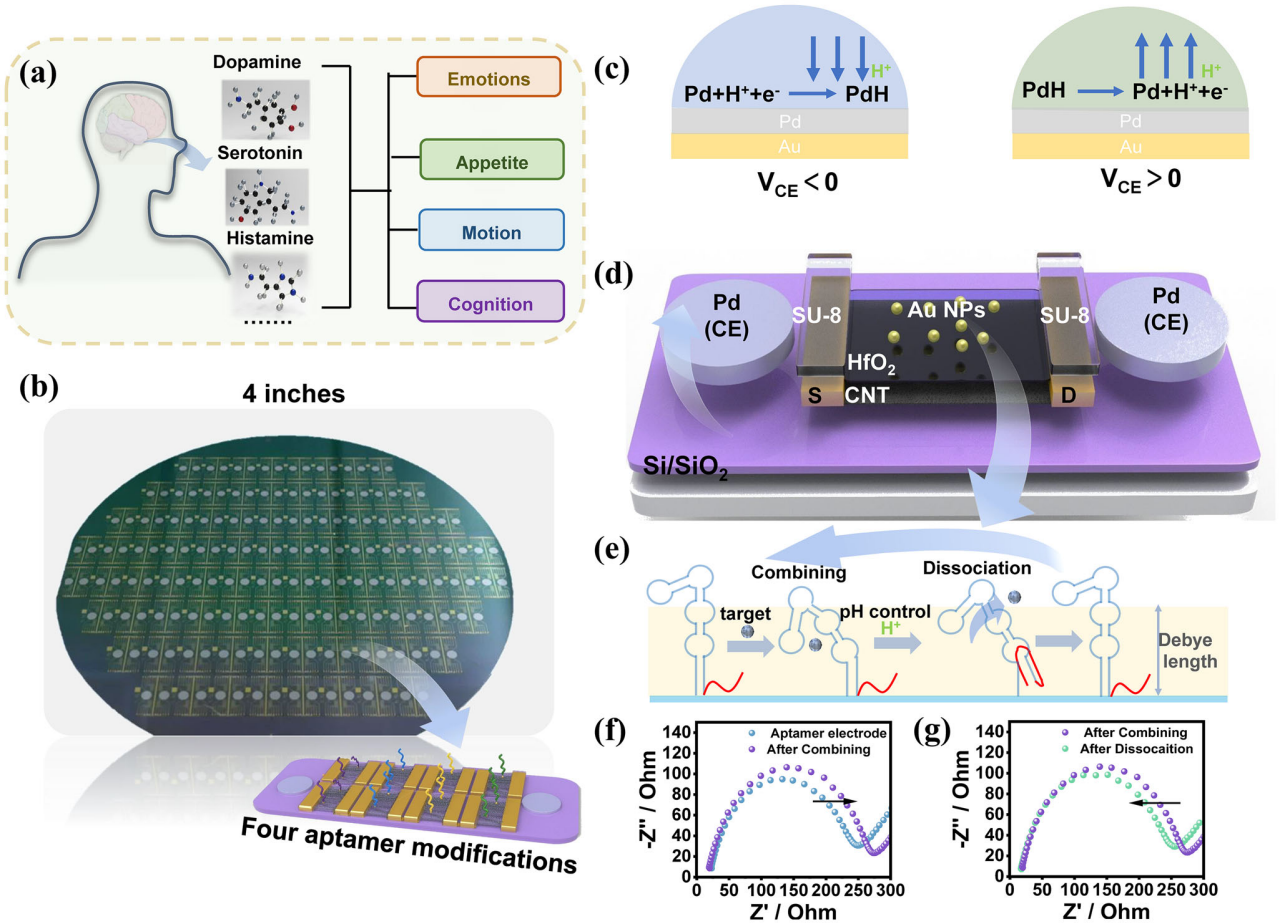
Resettable CNT FET biosensor arrays. a) Effects of neurotransmitters on human physiological functions, including regulation of mood, memory, and motor control. b) Photograph of CNT FET sensor arrays fabricated on a 4‐inch wafer. c) On‐chip CE for localized pH regulation, electrochemically driven to change the hydrogen ion concentration in the nearby environment. d) Schematic of the structure of a single sensor. e) Working principle of pH‐induced regeneration of sensing interface, where localized pH changes can lead to reversible recovery of the sensor. f) Sensor detection in PDR processes: Electrodes modified with aptamers are subjected to changes in the amount of surface charge after the aptamer binds to the target, resulting in a bigger impedance. g) Sensor recovery in PDR processes: Under acidic conditions, the affinity of the aptamer for the target decreases, leading to the release of the target and a shift in electrode impedance toward the initial state.

Neurotransmitters, including dopamine, serotonin, acetylcholine, glutamate, and γ‐aminobutyric acid (GABA), are essential for neurotransmission, regulating key physiological processes such as mood, memory, motor control, and cognition(**Figure** **1**a).^[^ ^1^, ^2^, ^3^, ^4^ ^]^ Dysregulated neurotransmitter levels are implicated in neurological disorders such as Parkinson's disease, depression, Alzheimer's disease, and epilepsy.^[^ ^5^, ^6^, ^7^, ^8^, ^9^ ^]^ Therefore, continuous monitoring of neurotransmitter levels is essential for understanding dynamic biochemical processes and for the wearable or implantable timely detection of fluctuations that may signal the onset of neurological disorders.

Traditional methods for neurotransmitter detection, such as high‐performance liquid chromatography (HPLC), electrochemical assays, and fluorescence‐based techniques, offer high accuracy and sensitivity.^[^ ^10^, ^11^, ^12^, ^13^, ^14^ ^]^ Nevertheless, these approaches typically necessitate molecular labeling (e.g., redox or fluorescent markers), involve laborious sample pretreatment protocols, and rely on sophisticated instrumentation, fundamentally limiting their applicability for continuous monitoring scenarios. In contrast, integrated carbon nanotube (CNT) field‐effect transistor (FET) biosensors can effectively transduce molecular recognition events into quantifiable electrical signals via modulating carrier concentration in the semiconducting channel, providing significant advantages especially including a high sensitivity and a label‐free detection ability.^[^ ^15^, ^16^, ^17^, ^18^ ^]^

Although CNT FET biosensor has great potential, its sensing process still relies on the affinity interactions between biological receptor molecules and targets.^[^ ^19^ ^]^ The strong affinity of these biological molecules, characterized by low dissociation constants, necessitates additional processing to facilitate rapid dissociation of probe and target molecules, which brings challenges to the reusability and real‐time monitoring of CNT FET biosensors. Existing strategies for accelerating biological probe‐target separation often require harsh surface treatments such as UV irradiation^[^ ^20^, ^21^ ^]^ or exposure to high concentrations of chemical reagents,^[^ ^22^ ^]^ which may damage the intrinsic device properties and structural integrity of the sensor. Moreover, the above methods require the introduction of external reagents or conditions to reconfigure the sensing interface, constraining applications to ex situ settings and making them difficult to integrate into portable or continuous in vivo monitoring systems. In addition, the nonspecific adsorption and sensor contamination caused by these operations limit their long‐term stability.^[^ ^23^ ^]^

To overcome these challenges, recent advances have focused on novel strategies to enhance sensor reusability without sacrificing performance. Examples of strategies for repeatable detection capabilities include the use of high‐frequency electric fields to facilitate probe‐target separation and the development of innovative sensing interfaces.^[^ ^24^, ^25^ ^]^ These approaches aim to improve signal‐to‐noise ratios and enable more precise targeting of biomolecules in complex environments. However, the integration of high‐frequency signals or the redesign of sensor interfaces often introduces external forces or new materials, which in turn impose higher demands on the stability of the probes and the precision of the detection system. One promising approach involves the conformational modulation of triple‐nucleic acid helices.^[^ ^26^, ^27^, ^28^, ^29^ ^]^ This conformational switching mechanism presents a viable solution for improving the reusability of FET‐based biosensors, ensuring reliable and reproducible performance over multiple detection cycles. A simple integrated reset method needs to be designed without damaging the intrinsic performance of the sensor.

Here, we present a resettable, multi‐target FET biosensor array based on CNTs designed to detect neurotransmitters at femtomolar (fM) concentrations with high sensitivity and specificity (Figure 1b). To achieve rapid probe‐target dissociation, we used pH‐sensitive aptamers as the probes. Through precisely modulating the potential of on‐chip Pd electrode, the biosensor facilitates in situ pH modulation and recovery of the sensor interface, which enables an electrically resettable biosensor. The as‐fabricated sensor arrays demonstrated the ability to in vitro detect dopamine, serotonin, histamine, and glutamate with a practical detection limit of 10 fM. Additionally, through in situ electrode modulation, the sensor arrays were successfully reused ten times with a response variation of less than 10%, demonstrating its well stability. The ability to monitor neurotransmitter dynamics in real‐time and in complex biological environments has significant implications for both neuroscience research and clinical diagnostics. Our work represents a step toward the development of a versatile, high‐performance platform for multi‐target biosensing that can advance our understanding of neurotransmitter functions and facilitate the early diagnosis of neurological disorders.

## 2 Results and Discussion

### 2.1 Design Concept of Resettable Probe

In conventional biosensors, target specificity is achieved by immobilizing probes on the sensing interface. Repetitive detection, however, typically requires additional chemical or physical processes to dissociate bound targets and regenerate the sensor's activity. Here, we leverage the metastable modulation of three‐stranded nucleic acid helices to dynamically control ligand‐receptor affinity. This approach employs DNA sequences capable of forming inter‐ or intramolecular triplex structures in acidic environments through Hoogsteen interactions. The design features two self‐complementary sequences separated by a loop, enabling self‐assembly into intramolecular triplex structures via parallel Watson–Crick and Hoogsteen base‐pairing (e.g., CGC^+^/TAT). For CG‐rich sequences, triplex formation is energetically favored under acidic conditions due to the protonation of cytosine at the N3 site, which facilitates Hoogsteen interactions. It has been shown that non‐affinity structural regions also affect the affinity and stability of aptamers by tuning the secondary structural of the entire aptamer.^[^ ^30^, ^31^ ^]^ At lower pH values, the assembly of intramolecular triplexes can disrupt the stem‐like structure of the aptamer or affect the affinity sequence of the aptamer, thereby releasing the target. Conversely, at higher pH, triplex unfolding restores the aptamer's target‐binding capability due to the absence of stabilizing hydrogen bonds.

Based on this concept, we designed an in situ resettable CNT FET biosensor utilizing pH modulation. A schematic diagram of a single sensor unit is shown in Figure 1d. A pH‐sensitive sequence (CG‐rich fragments) was introduced at one end of the original aptamer, which can form an intramolecular triplex with the stem through parallel Watson‐Crick and Hoogsteen interactions under acidic conditions very quickly and restore the free state when the pH increases. Our design was informed by molecular beacon design,^[^ ^30^ ^]^ where we chose CG‐rich aptamer fragments as the parent sequence and designed them with their cyclic double‐stranded fragments to facilitate control of the aptamer's structure by pH. The pH‐dependent domain is introduced in a location distal from the recognition sites in the aptamer. This allows to finely regulate the binding affinity of these DNA‐based aptamers with pH without affecting on the affinity of the recognition sequence of the original aptamer (Figure S1, Supporting Information). By incorporating pH‐sensitive aptamers, we enable the reversible release of bound ligands within seconds (Figure 1e).^[^ ^30^, ^32^, ^33^ ^]^ To achieve in situ regulation of pH, we electrically controlled the release and storage of H^+^ and thus the local pH values by applying a suitable voltage to an integrated Pd electrode. The Pd electrode was placed close to the sensing interface that can reversibly and locally control the pH value of the microenvironment while ensuring a minimal perturbation to surrounding areas (Figure 1c). This design enables repeated detection cycles without compromising the sensor's functionality. Importantly, our approach enables in situ resetting of the biosensor without the need for additional chemicals or external interventions. Electrochemical impedance spectroscopy (EIS) was performed to evaluate the impedance characteristics of the aptamer‐modified electrode. The measurements were conducted in a 0.1 × phosphate buffer solution (PBS, pH 7.4) containing 10 mm potassium ferricyanide (K₃[Fe(CN)₆]) as the redox probe. The frequency range was set from 100 kHz to 0.01 Hz with an AC amplitude of 5 mV at the open‐circuit potential. The Nyquist plots were analyzed to assess the charge transfer resistance (R_ct_) of the electrode before and after aptamer modification. Upon introduction of the target molecule at the electrode surface, a significant increase in R_ct_ was observed, indicating the occurrence of a specific peptidoglycan‐target binding event. The change in R_ct_ originated from a target‐induced conformational change in the aptamer structure, which hindered the electron transfer at the electrode surface (Figure 1f). Subsequent introduction of an acidic environment to the electrode surface resulted in a reduction of the aptamer‐target binding affinity. This led to partial dissociation of the aptamer‐target complexes and restoration of some aptamers to their original conformational state, with a corresponding partial recovery of R_ct_ to its initial level (Figure 1g). These results clearly demonstrate that the incorporation of pH‐sensitive fragments effectively enables the reconstruction of the sensing interface. The pH‐driven Recovery (PDR) highlights the reversible and dynamic nature of the aptamer‐target interaction, enabling the FET sensor to serve as a responsive and reusable detection platform.

### 2.2 Fabrication and Characterization of the CNT FET Arrays

**Figure 2:**
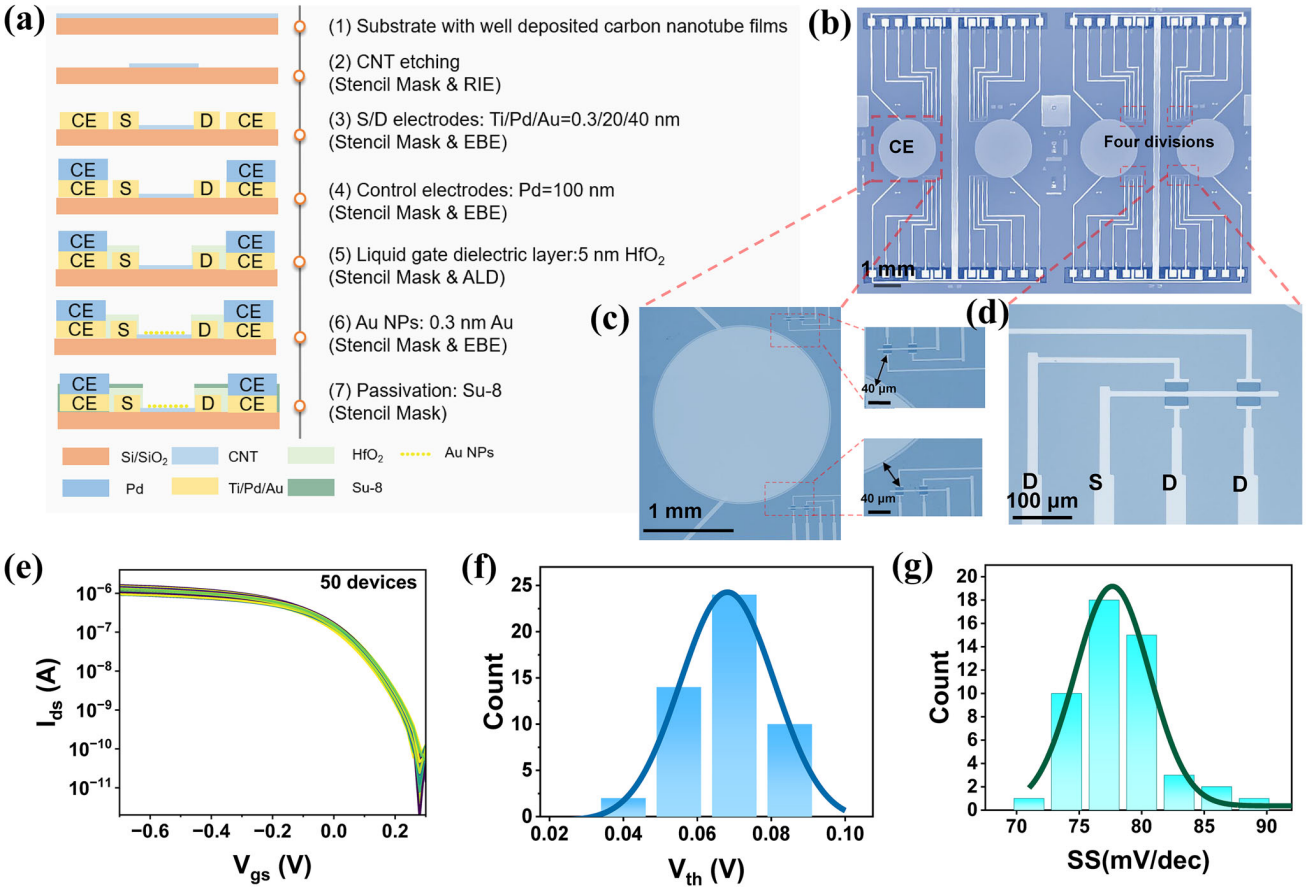
Fabrication process and characterization of CNT FET biosensor arrays. a) Flowchart of the preparation of the sensor arrays. b) Micrograph of two random sensing arrays on a 4‐inch wafer and a further enlarged micrograph of a portion of the channel region, where the sensing arrays are divided into four 4 × 4 regions to facilitate subsequent subregion modification. c) (I)Further enlarged optical micrograph showing the CE region of the biosensor array. (II) and (III) Additional magnified views highlight a 40 µm spacing between the CE and the sensor region. d) Optical micrograph of FET biosensors, where the metal contacts and leads are passivated by a photoresist layer. e) Transfer curves of 50 FETs at *V*_ds_ =‐0.1 V.f,g) Histograms of the statistical distributions of *V*_th_ and SS based on the batches of 50 FET devices in Figure 2e, all of which conform to a normal distribution.

Polymer‐sorted and solution‐derived single‐walled CNTs were deposited onto 4‐inch Si/SiO₂ wafers to achieve semiconductor‐grade purity (> 99.99%),^[^ ^34^ ^]^ forming uniformly distributed and randomly oriented films. The Raman characterization of CNTs is shown in Figure S2 (Supporting Information), which proves that the film of CNTs deposited is excellent. Subsequently, CNT FET biosensor arrays were fabricated on the wafers using photolithography (**Figure** **2**a). The FETs were constructed with HfO₂, grown by atomic layer deposition (ALD), as the gate dielectric on the CNT films. The photographs in Figure 2b show biosensor array chips, each containing 16 FETs. The exposed channel region was decorated with Au nanoparticles(Au NPs), which were uniformly distributed on HfO₂ and served as an effective linker to connect the biological probes (Figure S3, Supporting Information).^[^ ^35^, ^36^ ^]^ The symmetrical design of the sensor array, with pH adjustment electrodes on both sides, ensures consistent regulation of the four sensing zones. The circular pH CE is 2 mm in diameter and was designed to be located 40 µm from the sensor to maximize the impact on the sensing interface (Figure 2c). The sensor contacts and leads were passivated by a photoresist layer to prevent 1) current leakage in the ionic liquid, 2) lateral electrochemical reactions, and 3) power function modulation of the metal contacts and leads, while a 20 µm × 40 µm window was exposed in the channel region (Figure 2d). For reliable and accurate bioassays, biosensors must exhibit high consistency and stability. In performance verification, transmission characteristics of 50 randomly selected CNT FET devices were assessed. The current on/off ratios of all devices reached ≈10⁵ with minimal variation (Figure 2e). This establishes the foundation for stable, large‐scale applications. Stability tests further demonstrated that a randomly selected FET device exhibited highly overlapping curves, with current fluctuations of less than 6% across 10 transmission curve tests. In particular, the hysteresis‐free and low subthreshold slope (SS) of 80 mV dec^−1^, indicating stable response uniformity and sensitivity during repeated testing. The sensor drift was minimal during 5 min of real‐time testing with a sensor four‐device blank (Figure S4, Supporting Information). The statistical performance distributions in Figure 2f,g confirm the uniformity of CNT FETs: The mean threshold voltage (*V*_th_) was 0.06 V with a standard deviation of 10 mV, and the mean SS was 78 mV dec^−1^ with a standard deviation of 3.33 mV dec^−1^, demonstrating the high consistency and repeatability of the devices. The excellent stability will promote CNT FET biosensors in long‐term continuous monitoring applications.

### 2.3 CNT FET Biosensor Arrays for Single Neurotransmitter Detection

**Figure 3:**
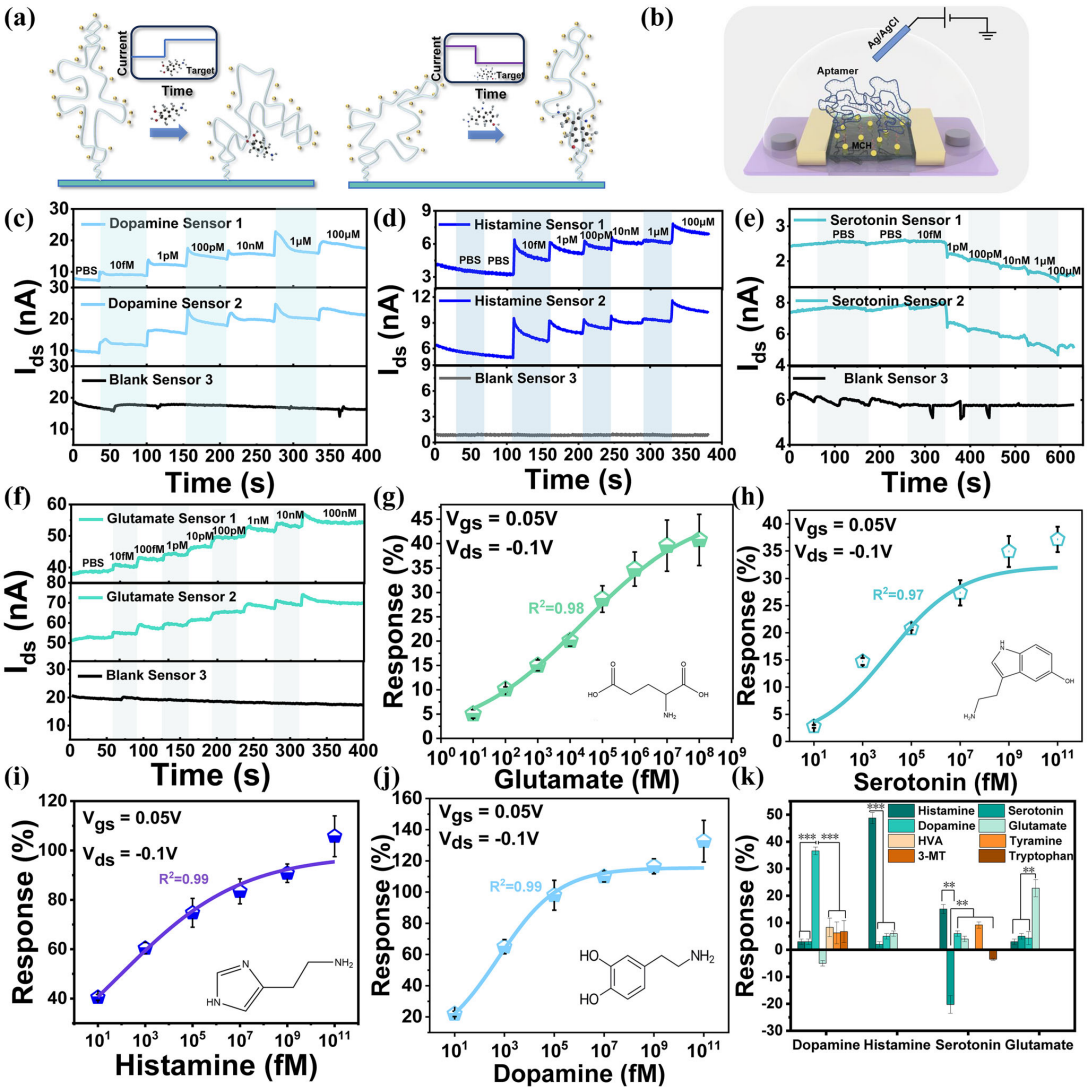
CNT FET biosensor for detecting a single neurotransmitter assay. a) Schematic illustration of the aptamer‐based FET biosensor detection mechanism. Target binding induces a conformational change in the aptamer, resulting in modulation of the sensor's current level. b) Schematic representation of sensor detection configuration. The aptamer was immobilized on the channel surface, with excess Au‐S sites passivated using MCH, and an Ag/AgCl reference electrode was introduced. c) Three‐channel simultaneous real‐time *I*_ds_ measurements comparing biosensors with and without dopamine aptamers, demonstrating response to dopamine concentrations ranging from 10 fM to 100 µM in 0.1× PBS. d) Three‐channel simultaneous real‐time *I*_ds_ recordings of histamine aptamer‐functionalized and control biosensors, showing responses to histamine concentrations from 10 fM to 100 µM in 0.1× PBS. e) Real‐time three‐channel *I*_ds_ measurements of serotonin aptamer‐modified and control biosensors upon addition of serotonin at concentrations ranging from 10 fM to 100 µM in 0.1× PBS. f) Simultaneous three‐channel real‐time *I*_ds_ recordings of glutamate aptamer‐functionalized and control biosensors responding to glutamate concentrations from 10 fM to 100 nM in 0.1× PBS. g). Calibration curves of glutamate aptamer‐modified sensors detect different concentrations of glutamate. h) Calibration curve of serotonin aptamer‐modified sensors detecting different concentrations of serotonin. i) Calibration curve of histamine aptamer‐modified sensors for detection of different concentrations of histamine. j) Calibration curve of dopamine aptamer‐modified sensors for different concentrations of dopamine. k) Specificity assessment of the sensor array. Measurements were performed with dopamine, serotonin, histamine, and glutamate at 1 pM, and all potential interferents at 1 nM. All error bars represent the standard deviation of the results from three parallel experiments. n = 3, ^**^ *p* < 0.01, ^***^ *p* < 0.001. All data presented as mean ± SD.

Au NPs are decorated on the HfO₂ layer of CNT FETs array and act as linkers to capture sulfhydryl probe molecules by Au−S covalent bonds (**Figure** **3**b). The CNT/ HfO₂/ Au NPs stacked layer forms the FET sensing platform. In order to investigate the conformational changes and affinity upon aptamer binding, we tested the conformational changes of the four aptamers in Table S1 (Supporting Information) upon binding to the targets in a distributed manner. All aptamers produced significant conformational changes upon binding to the targets (Figure S5, Supporting Information). We systematically characterized the affinities of the aptamers using the surface plasmon resonance (SPR) technique. As shown in Figure S6 (Supporting Information), the screened aptamers all exhibited excellent binding affinities with K_d_ values of dopamine (20 nm), serotonin (10.5 nm), histamine (15 nm), and glutamate (293 nm). To selectively detect specific neurotransmitters, the probe DNA sequences are immobilized by the Au NPs, and then the neurotransmitter biosensor is ready. The transfer characteristics of the CNT FET before and after modification are presented in Figure S7 (Supporting Information). The modified CNT FET exhibits a 60 mV *V_th_* shift in the *I*_ds_–*V*_gs_ curve, indicating p‐type electrostatic doping induced by the negative charge gating of the aptamer. Upon introduction of the target molecule to the sensor surface, the aptamer undergoes target‐specific binding accompanied by a conformational reorganization, thereby modulating the local charge distribution near the sensing interface. When the new conformation moves closer to the sensing surface, it attracts more positive charges due to electrostatic interactions, resulting in a rightward shift in the *V*_th_ and an increase in the p‐type FET current. Conversely, when the conformation moves farther away from the sensing surface, the opposite effects are observed (Figure S8, Supporting Information). These results confirm that the aptamers have been successfully immobilized on the HfO₂ and can bind to target molecules. The resulting charge transfer is attributed to conformational changes induced by the aptamer‐target binding event (Figure 3a).

To evaluate the performance of the biosensor array, we tested four neurotransmitters: dopamine, serotonin, histamine, and glutamate. The model for single‐target detection is shown in Figure 3b. The Ag/AgCl reference electrode is used to apply the liquid gate to stabilize the potential of the electrolyte, while the back gate is grounded. The response data were extracted from the real‐time test curves. The sensor response is typically defined as follows: at specific values of *V*_ds_ and *V*_gs_, the response = (|*I*_ds_‐*I*_ds0_|) / *I*_ds0_, where *I*_ds0_ and *I*_ds_ represent the drain current before and after target molecule capture. For real‐time testing, we aimed to operate the sensor in the subthreshold region to maximize the sensing response. Based on previous statistical results, we set *V*_ds_ to −0.1 V and *V*_gs_ to 0.05 V. The test design utilized a split‐region modification approach, where on a single chip, some sensors were functionalized while the remaining unmodified sensors served as blank controls. A triplicate sensor configuration was employed for simultaneous measurements, consisting of two aptamer‐functionalized sensors and one unmodified control sensor. Conducting control experiments on the same chip minimizes the interference of non‐specific adsorption on the experimental results, thereby reducing the likelihood of false positives to the greatest extent possible. We extracted the responses from the real‐time measurements and fitted the response‐concentration curves to the Langmuir isothermal adsorption model (Figure 3c–j). We take the average of the two sensor responses for statistical purposes, which makes our responses more reliable and facilitates quantitative analysis. The response curves closely followed the Langmuir adsorption isotherm, and the actual limit of detection (LOD) of the sensor for the four neurotransmitters in a buffered environment was 10 fm. The sensor arrays have a detection range spanning eight orders of magnitude or more for all four neurotransmitters, fully meeting the requirements for neurotransmitter detection.^[^ ^37^ ^]^

To verify the accuracy and specificity of the sensor response, we tested the sensor's resistance to interference and performed parallel statistical analyses. We statistically analyzed the responses of the target samples and the various interferent samples to the sensor using a paired‐sample *t*‐test, which showed significant differences between the groups of interferent and target samples at *p* < 0.001 for dopamine and histamine and *p* < 0.01 for serotonin and glutamate. The results clearly showed that the sensors could specifically differentiate between different targets (Figure 3k). Negative control tests using unmodified sensors were also performed. For the unmodified sensor arrays, the current returned to the baseline level after a transient fluctuation induced by the addition of 0.1× PBS, and the change in current after introducing the target molecule was minimal (less than 5%). These findings demonstrate the excellent specificity of the sensor arrays (Figure S9, Supporting Information).

### 2.4 PDR CNT FET Biosensor Arrays for Reproducible Detection

**Figure 4:**
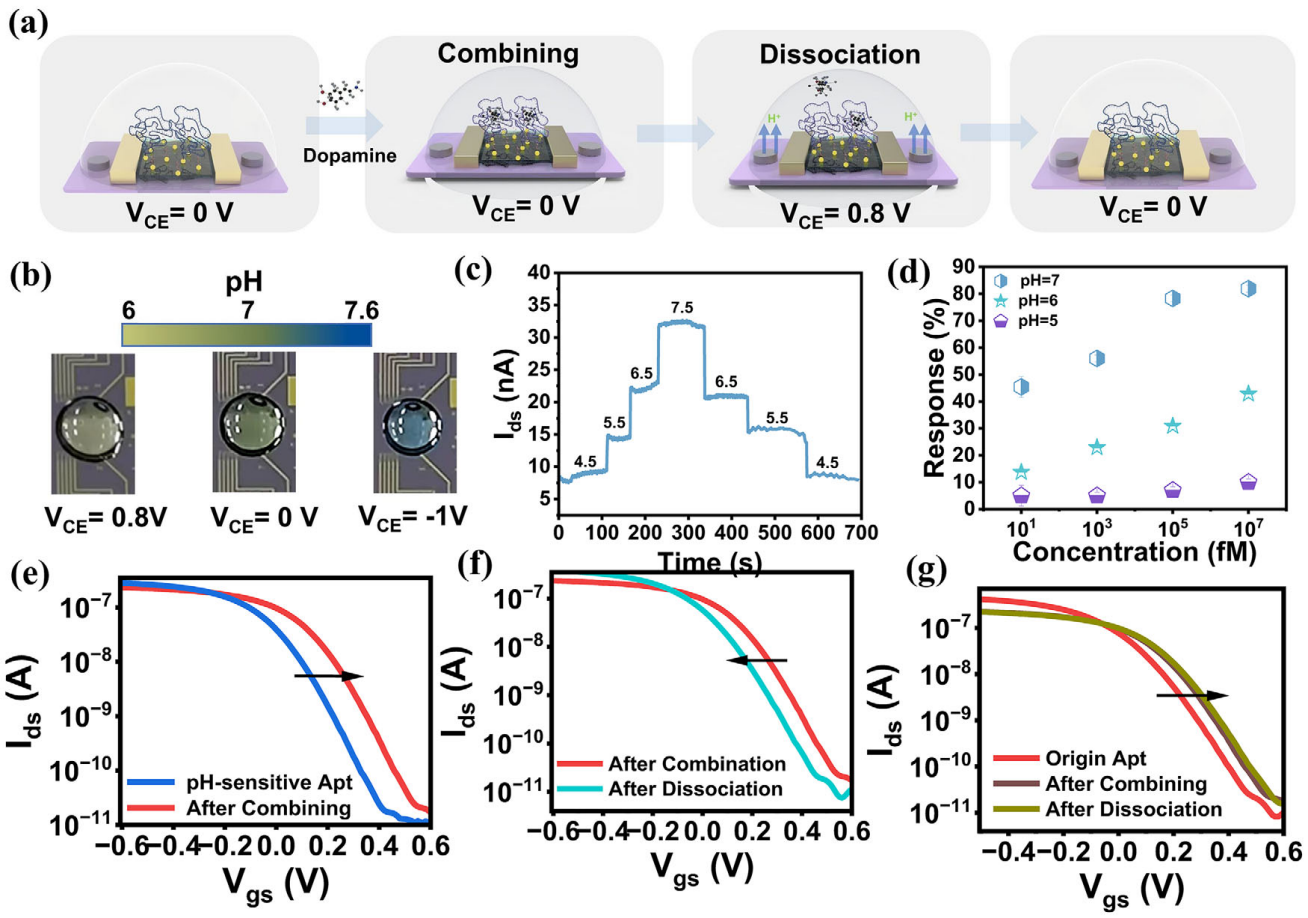
PDR process and repeatable detection. a) Schematic diagram of the PDR process. At *V*_CE_ = 0 V, the probe and target exhibit strong affinity, enabling normal assembly. When *V*_CE_ is increased to 0.8 V, an electrochemical reaction occurs on the surface of the CE, releasing H⁺ ions. This leads to a structural change in the aptamer, resulting in the release of the bound target. Upon resetting *V*_CE_ to 0 V, the aptamer structure is restored, allowing it to recapture the target. b) color reaction of PBS Containing Bromothymol Blue at different *V*_CE_ values. As *V*_CE_ decreases, the droplets on the CE surface exhibit a more bluish color, indicating a gradual increase in solution pH. c) Recoverability of the FET biosensor with HfO_2_ as the gate medium for pH effects. The *I*_ds_ changes in response to variations in environmental pH. However, when the pH returns to its initial level, the *I*_ds_ also reverts to its original value, highlighting the sensor's recoverability. The measurement was performed at *V*_ds_ = −0.1 V and *V*_gs_ = 0.05 V. d) Tabulation of pH‐sensitive aptamer target capture ability at different pH values. The error bars represent the standard deviation of the results from three parallel experiments. The response of the sensor to dopamine concentrations (ranging from 10 fM to 10 nM) diluted in background solutions of varying pH was tested. The results demonstrate the pH‐dependent target capture efficiency of the aptamer. n = 3, data presented as mean ± SD. e) FET Sensor detection in PDR processes: The CNT FET exhibited a *V*_th_ shift in the *I*_ds_‐*V*_gs_ curve, while no significant changes were observed in transconductance (g_m_) or SS. These results suggest p‐type electrostatic doping induced by the negative charge gating effect of the aptamer. f) FET Sensor Recovery in PDR Processes: Under acidic conditions, the aptamer separates from the target, resulting in a shift in the opposite direction of the *V*_th_ value on the *I*_ds_‐*V*_gs_ curve of the CNT FET. g) Changes during PDR with sensors modified with aptamers that do not introduce pH‐sensitive fragments. The sensor detects the target but does not enable sensing interface recovery. All transfer curves were measured at *V*_ds_ = −0.1V.

As previously mentioned, the PDR process requires the addition of a pH‐sensitive sequence to the 3' end of the aptamer, which induces a conformational change from a double‐stranded to a triple‐stranded structure in an acidic environment. **Figure** **4**a illustrates the regeneration process, where transformation is driven by pH regulation. Under neutral pH conditions, the aptamer adopts its native conformation and maintains a strong affinity for the target, allowing for normal target binding. The sensor response is reflected by changes in surface charge, which result in variations in the source‐drain current. Upon applying a positive voltage to the Pd electrode, deprotonation occurs on the Pd surface, leading to a decrease in the local pH of the solution. This, in turn, triggers the aptamer conformational change, weakening its interaction with the target, and releasing the bound ligand.

For demonstration purposes, we used our sensor arrays to detect dopamine. By adding a pH‐sensitive sequence to the 3' end of the dopamine aptamer, the aptamer's affinity for dopamine is modulated by pH (Figure S1, Supporting Information). The functionality of the pH regulator was tested by exposing the Pd electrode to proton electrochemistry in an acidic environment, followed by release tests in a neutral pH. The electrode was immersed in a 500 mm NaCl solution containing 0.1 mm H₂SO₄ at pH 4.0, with *V*_CE_ = −1.0 V for 300 s. This allowed the adsorption and reduction of protons (H^+^), forming PdHx. Afterward, the hydrogen‐loaded electrode was transferred to a 0.1× PBS at pH 7.4, with *V*_CE_ = 0.8 V, leading to proton oxidation and subsequent release (Figure S10, Supporting Information). The calculated total amount of proton capacity of the electrode is ≈6.02 × 10^−7^ mol (Note S1, Supporting Information). To evaluate the effect of the electrode on local pH at various *V*_CE_ values, we introduced a pH indicator (bromothymol blue) into the PBS (Figure 4b). The droplets on the Pd electrode exhibited distinct color changes, corresponding to different pH values due to the varying electrode reactions at different *V*_CE_ values, which regulate the capture and release of H^+^. To ensure that local pH changes near the sensor's channel do not cause irreversible effects, we tested the sensor's resistance to pH fluctuations (Figure 4c). The results indicated that the pH variation had a negligible impact on the sensor. Previous work has demonstrated that CNT‐based sensors using HfO_2_ show good pH detection capabilities.^[^ ^38^ ^]^ To further validate our system, we tested the liquid environment using a HfO_2_‐based FET device, calibrated with a standard pH solution. The pH regulation range was determined to be between 5 and 8 (Figure S11, Supporting Information), confirming that the Pd electrode can effectively induce local pH changes.

Having confirmed the capability of the Pd electrode to regulate pH, we proceeded to examine the affinity of pH‐sensitive aptamers for the target at various pH levels (Figure 4d). When the detection environment was neutral, the aptamer exhibited a strong binding affinity for the target. However, as the pH decreased, the sensor response gradually diminished, indicating a reduction in the aptamer's affinity for the target. This provides strong evidence that PDR can be effectively utilized to achieve repeatable detection with FET sensors.

We conducted additional experiments using the FET sensor by functionalizing the sensing interface with a pH‐sensitive dopamine‐specific aptamer. The modified sensor was subsequently challenged with dopamine at a concentration of 1 nm. Under neutral pH conditions, the aptamer demonstrated successful binding to its target dopamine molecules, confirming the functionality of the sensing platform. This change induces electrostatic gating effects, resulting in a measurable shift in the *V*_th_ of the FET sensor (Figure 4e). In contrast, under acidic conditions, the aptamer transitions from a double‐stranded to a triple‐stranded conformation. As a result, the charge distribution near the sensing interface returns to its original state, and the V*_th_* value of the FET sensor shifts back toward the baseline level (Figure 4f). The sensor's baseline is difficult to recover to the initial level due to non‐specific adsorption, but this interference is significantly reduced in subsequent tests. We performed the PDR process on aptamer‐modified sensors that did not introduce pH‐sensitive fragments. The sensors were able to achieve normal detection of the target substance, but the reconfiguration of the sensing interface could not be realized (Figure 4g). This proves that our redesign of the probe is effective in enhancing the repeatability of the sensor.

### 2.5 Reusable In Vitro Multiplexed Assays for Neurotransmitters

Simultaneous multi‐target detection presents significant challenges, primarily due to direct crosstalk between functionalized regions and the risk of non‐specific adsorption, which can lead to inaccurate or unreliable sensor responses. Sensor arrays were developed to enable the concurrent detection of dopamine, serotonin, histamine, and glutamate on a single sensor chip. Benefiting from the sensor array design as shown in Figure 2b, we achieved the modification of four probes in different regions on the same chip. Specifically, the sensor array was divided into four distinct regions, each functionalized with unique aptamers targeting specific neurotransmitters. Specific aptamers were immobilized in spatially distinct regions of the biosensor, thus minimizing the interference while allowing testing with shared biological samples (Figure S12, Supporting Information). The multi‐target testing system, as depicted in Figure S13 (Supporting Information), was further enhanced by encapsulating polydimethylsiloxane (PDMS) microchannels on the sensor surface to precisely control the test environment. This design allows for the continuous removal of excess reagents, significantly reducing crosstalk and non‐specific interactions. Additionally, a direct current (DC) power supply was employed to power the on‐chip Pd electrode, enabling subsequent PDR testing.

**Figure 5:**
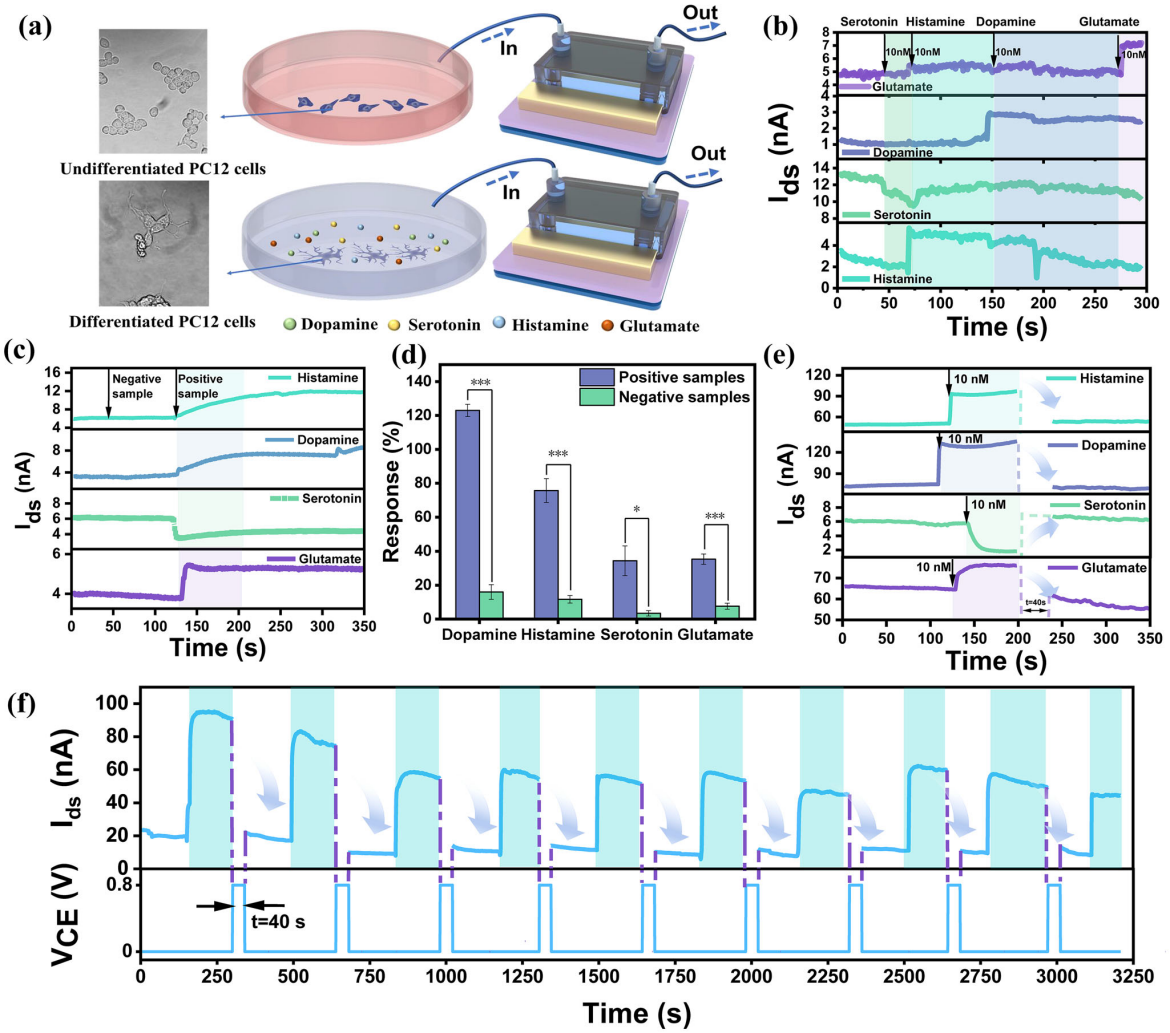
Reproducible in vitro multiplexed assays for neurotransmitters. a) Schematic illustration of the in vitro experimental for detecting multiple neurotransmitters (dopamine, serotonin, histamine, and glutamate) released by PC12 cells using a sensor array. b) Interference resistance test of the culture medium: Current‐time curves obtained from the simultaneous detection of four neurotransmitters in PC12 cell‐specific culture medium using the sensor array. A sample solution containing only one target analyte is introduced at a time. After the current stabilizes, the next sample is introduced sequentially. The analytes tested include 10 nM serotonin, 10 nM histamine, 10 nM dopamine, and 10 nM glutamate. c) Neurotransmitter secretion assay in nerve cell samples. Negative controls comprised undifferentiated nerve cells lacking neurotransmitter secretion capability, whereas positive samples consisted of fully differentiated, functionally active nerve cells exhibiting normal neurotransmitter secretion. All samples were tested by tenfold dilution with culture medium. d) Response plots for in vitro sensing assay of PC12 cell samples: The values of the current changes before and after the addition of the samples were extracted from the curves in Figure 5c and the responses were calculated. All error bars were taken from four sets of parallel experiments. n = 4, ^*^ *p* < 0.05, ^***^ *p* < 0.001. Data presented as mean ± SD. e) pH‐sensitive aptamer‐modified FET sensors are reproducibly tested. After the introduction of the target, the sensor responds to the target, and the sensing interface is reconfigured by the PDR process with the I–t curve current returning to baseline. f) The Real‐time curve of the sensor over ten repeated cycles. A 10 nm dopamine solution diluted in 0.1× PBS (pH 7.4) was introduced onto the sensing surface at *V*_CE_ = 0 V, and real‐time current measurements were performed. After the current stabilized, *V*_CE_ was set to 0.8 V for 40 s to separate the aptamer from the target, followed by resetting to 0 V. This cycle was repeated ten times, demonstrating the sensor's repeatability and stability. All measurements were performed under the conditions of *V*_ds_ = −0.1 V and *V*_gs_ = 0.05 V.

To validate the monitoring capability of the sensor in complex biological matrices that mimic the in vivo environment, this study employed the sensor to detect the release of multiple neurotransmitters from PC12 cells. The differentiation of PC12 cells induced by nerve growth factor (NGF) represents a classical model for investigating neurotransmitter release. In their undifferentiated state, PC12 cells predominantly exhibit endocrine cell characteristics, capable of synthesizing neurotransmitters but lacking the ability to release them. Following NGF treatment, PC12 cells differentiate into neuron‐like cells after 48 h of culture, acquiring the capacity to synthesize and release multiple neurotransmitters (**Figure** **5**a). To eliminate potential interference from the culture medium on the detection of neurotransmitter release, we first conducted control experiments by spiking PC12‐specific culture medium with known concentrations of dopamine, serotonin, histamine, and glutamate, followed by detection using the sensor array (Figure 5b). The sensor array exhibited distinct responses to dopamine, serotonin, histamine, and glutamate, with response values of 130%, 20%, 109%, and 47%, respectively (Figure S14, Supporting Information). Notably, the sensor array demonstrated negligible cross‐reactivity to the other three neurotransmitters in their respective detection regions, indicating its high specificity for neurotransmitter detection even in the complex environment of the culture medium. Subsequently, we performed in vitro detection of neurotransmitter release from PC12 cells. Culture media were collected from undifferentiated PC12 cells (serving as a negative control) and NGF‐induced differentiated PC12 cells (serving as a positive sample) after 48 h of growth. The concentrations of four neurotransmitters—dopamine, serotonin, histamine, and glutamate—in the collected media were then analyzed using the sensor array. This method effectively simulates neurotransmitter release under physiological conditions, thereby robustly demonstrating the capability of the sensor array for detecting neurotransmitters in real‐world biological samples. The experimental results are presented in Figure 5c,d. Figure 5c illustrates the real‐time detection profiles of the sensor array for the four neurotransmitters released by both undifferentiated and NGF‐induced differentiated PC12 cells. Data from four independent replicate experiments were extracted and analyzed, as depicted in Figure. 5d. The sensor array exhibited distinct responses to neurotransmitters in the positive samples, with response values of 122.9%, 75.6%, 34.3%, and 35.3% for dopamine, histamine, serotonin, and glutamate, respectively. We statistically analyzed the responses of negative and positive samples for various neurotransmitters using paired‐samples t‐tests with p< 0.001 for dopamine, histamine, and glutamate, and p< 0.05 for serotonin, which indicated significant differences between negative and positive samples in each group. Control samples demonstrated minimal interference with sensor performance, with the average non‐specific response remaining below 10% for all sensors except the dopamine sensor, which showed a slightly higher non‐specific response. However, this response was still below 20%, indicating negligible interference and confirming the high specificity of the sensor array under experimental conditions. The results underscore the potential of the sensor array as a reliable tool for studying neurotransmitter dynamics in complex biological environments. We then tested the real‐time response of our sensors with the PDR process. We modified each of the four pH‐sensitive neurotransmitter probes on each of the four microarrays using the modification method described previously. *V*_ds_ was set to −0.1 V and *V*_gs_ was set to 0.05 V. The sensors responded significantly after the introduction of 10 nm of the target at the sensing interface, and the sensor response stabilized after 200 s of real‐time testing. Afterward, an acidic environment was introduced for 40 s and the sensors were reset, and then the test was conducted by passing the background solution, and the current levels of all four sensors returned to baseline, providing ample evidence that the sensors can be reused (Figure 5e). During the pH regulation phase, the FET is turned off due to the +0.8V bias, pausing the signal reading.

Finally, we integrated a pH‐modulated electrode and systematically evaluated the sensor's performance stability across multiple operational cycles through repeated dopamine detection assays, as illustrated in Figure 5f. The *V_ds_* and *V_gs_* were set to −0.1 and 0.05 V, respectively. We modified pH‐sensitive dopamine aptamers on a sensor for repetitive testing. A 10 nm dopamine solution was then introduced to the sensor surface, which was in a background solution of 0.1× PBS at pH 7.4. At this point, the dopamine aptamer retained its native conformation, enabling specific binding to dopamine molecules. Upon binding, the aptamer underwent a conformational change, altering the charge distribution on the channel surface and thereby modifying the source‐drain current. To restore the sensor baseline, a +0.8 V bias was applied to the Pd electrodes for 40 s. After this treatment, real‐time current measurements revealed that the baseline current returned to its original value, effectively resetting the sensor to its pre‐binding state. Subsequently, another 10 nm dopamine solution was introduced to the sensor in the same background solution, resulting in a reproducible sensor's response. This process was repeated ten times, with each iteration yielding consistent and distinct responses, demonstrating the reproducibility and stability of the sensor. We homogenized the sensor response and extracted the standard deviation. σ = 0.05. The sensor response fluctuated over a range of ten percent (Figure S15, Supporting Information). The PDR reset mechanism facilitates the aptamer return to the unbound state within 1 min, which allows for continuous monitoring. The repeatability and reproducibility observed in this study underscore the potential of PDR as a promising strategy for preserving sensor performance over prolonged periods and multiple cycles. This capability is crucial for practical applications in areas such as environmental monitoring, clinical diagnostics, and wearable health sensors.

## 3 Conclusion

We have developed a multi‐target, resettable CNT FET biosensor array. In situ recovery of the sensing interface, as well as high sensitivity for simultaneous detection of multiple substances, was achieved by subregional functionalization of CNT FETs with specific pH‐sensitive aptamer probes and integration of on‐chip Pd electrodes. We achieved remarkable sensitivity (femtomolar detection limits), high selectivity (specific responses 20 times greater than non‐specific ones), and excellent reproducibility (stable response maintained over ten reuse cycles). Meanwhile, the introduction of the detection microfluidic channel technology reduces the effect of nonspecific adsorption. In in vitro assays with complex samples, the sensor specifically detected four neurotransmitters, validating its stability and reliability in complex biological environments. These findings mark significant progress in the design of reconfigurable biosensors for neurochemical analysis, which can be widely used in neuroscience research and clinical diagnosis. In addition, this approach is expected to be further optimized, laying a solid foundation for the development of advanced diagnostic tools capable of real‐time monitoring of various biochemical targets.

## 4 Experimental Section

### Materials

Phosphate‐buffered saline (PBS, pH 7.4, containing 137 mm NaCl, 2.7 mm KCl, 8.1 mm Na_2_HPO_4,_ and 1.76 mm KH_2_PO_4_) was purchased from Sangon Biotech (Shanghai, China). 6‐mercapto‐1‐hexanol (MCH) was purchased from Sigma‒Aldrich (Shanghai, China). Dopamine, histamine, glutamate, and tryptophan were purchased from Beijing XinXiYuan Biotechnology Co, Ltd (Beijing, China). Serotonin was purchased from Beijing Belo Biotechnology Co, Ltd (Beijing, China). 3‐Methoxytyramine (3‐MT) was purchased from Beijing Kepujia Experimental Instrument Co, Ltd (Beijing, China). Tyramine was purchased from Shanghai Dibao Biotechnology Co, Ltd (Shanghai, China). Homovanillic Acid (HVA) was purchased from Beijing Qisong Biotechnology Co, Ltd (Beijing, China).

### Aptamer Immobilization

First, the aptamer was diluted to a concentration of 2 µm in 1× PBS. This solution was then mixed with tris(2‐carboxyethyl) phosphine hydrochloride (TCEP) in equal volumes, and further diluted to 2 mm using 1× PBS, resulting in a final concentration of 1 µm aptamer solution. To facilitate the cleavage of disulfide bonds between aptamer molecules, the mixture was incubated at room temperature for 10 min. This step was essential for promoting the subsequent covalent attachment of the aptamer to Au NPs. After incubation, 30 µL of the activated aptamer solution was carefully applied dropwise to the channel region of the FET device, and the assembly was left to incubate for 12 h, allowing sufficient time for the formation of stable Au─S bonds between the aptamer and Au NPs, ensuring robust binding. After this incubation, the FET devices were cleaned sequentially with 1× PBS and deionized water to remove any excess aptamer that might have adhered nonspecifically to the FET channel surface. To further reduce nonspecific adsorption, 100 µm mercaptohexanol (MCH) was added dropwise into the trench, followed by a 30‐min incubation. After this step, the sensors were thoroughly rinsed with PBS and deionized water. The biosensor arrays were then ready for use, with any unused aptamers stored at 4 °C to maintain their stability.

### PDR Process

Following the modification and passivation of the sensor, the target analyte, diluted in PBS at pH 7.4, was introduced into the system while *V*_CE_ was maintained at 0 V. In this neutral environment, the aptamer exhibits a high affinity for the target, enabling efficient binding. The FET sensor detects the target analyte during this phase. Upon reaching binding saturation, a voltage of *V*_CE_ = 0.8 V was applied for 40 s. This voltage application triggers a deprotonation process on the surface of the CE, resulting in the release of H⁺ and a subsequent decrease in the local pH. Under these acidic conditions, the aptamer undergoes a conformational transition from a duplex to a triplex structure, which significantly reduces its affinity for the target analyte, leading to target release. The released target molecules were then flushed away by a blank background solution flowing through the microfluidic channel. By resetting *V*_CE_ to 0 V, the H⁺ release from the CE surface ceases, and the local pH returns to neutral. Consequently, the aptamer reverts to its duplex state and regains its binding capability for the target analyte in the neutral buffer. This completes one cycle of the PDR process, enabling the sensor to be reused for subsequent detection cycles.

### Electrical Measurement

All electrical measurements were conducted using a probe station. The transfer and output characteristics, as well as real‐time tests of the FET sensors, were performed with a Keithley 4200 semiconductor analyzer. For four‐channel testing, the PDA FS‐Pro was used to provide *V*_gs_, *V*_CE_, and *V*_GND_. For static tests, the target solution was diluted to the required concentration with 0.1× PBS and carefully drop‐cast onto the prepared biosensor channel. The sensor was incubated for 1 h to ensure complete binding between the sensor and the target molecules. After incubation, the sensor channel was rinsed with 0.1× PBS and deionized water to remove any non‐specifically adsorbed target molecules. Finally, a 30 µL drop of 0.1× PBS was added to the channel for transfer curve testing. Real‐time detection was performed with the *V*_ds_ set to −0.1 V. Prior to testing, an Ag/AgCl reference electrode (RE) was immersed in the 0.1× PBS solution to establish the *I*_ds0_). The target solution, diluted with 0.1× PBS, was then introduced.

### In Vitro Neurotransmitter Sensing Detection

Remove frozen PC12 cells from liquid nitrogen and thaw quickly in a 37 °C water bath. The cell suspension was transferred to a centrifuge tube containing complete medium and centrifuged at 1000 rpm for 5 min. Discard the supernatant, resuspend the cells with PC12‐specific medium, inoculate them into petri dishes, and place them in a 37 °C, 5% CO₂ incubator. The old medium was aspirated and replaced with PC12 cell special medium containing NGF 50ng/mL to induce cell differentiation. Fresh medium containing NGF was replaced every 2–3 days. The negative control group (undifferentiated group) was cultured with PC12 special medium without NGF. The cell morphology was observed periodically with an inverted microscope, and the growth of neurites was recorded by taking pictures. 48 h later, the media of both groups were centrifuged to ensure that the cells would not be extracted when extracting the subsequent extracted samples. The culture medium supernatants of the negative control group (undifferentiated group) and the positive group (differentiated group) were collected separately for spare use.

### Statistical Analysis

All information in this study was expressed as mean ± standard deviation (mean ± SD, n = 3). Data were analyzed using Origin (Origin, 2021, Origin Lab, USA), and results are shown as mean ± standard deviation (SD). Error analysis was performed by t‐test with at least 3 independent experiments for all error bars. Independent samples t‐tests were performed for between‐group comparisons with a sample size of at least n = 3 for each group. statistical significance was as follows: ^*^ *p* < 0.05, ^**^ *p* < 0.01, ^***^ *p* < 0.001.

## Conflict of Interest

The authors declare no conflict of interest.

## Supporting information



Supporting Information

## Data Availability

The data that support the findings of this study are available from the corresponding author upon reasonable request.
