# Supplementary material for: In Situ Electrically Resettable Field‐Effect Transistor Biosensors for Continuous and Multiplexed Neurotransmitter Detection
Source: Adv Sci (Weinh). 2025 May 28;12(31):e04497. doi: 10.1002/advs.202504497 (PMC12376627; doi:10.1002/advs.202504497)
Supplement: Supplementary file 1 — Supporting Information [file ADVS-12-e04497-s001.docx]

**Supporting Information**

**In-Situ Electrically Resettable Field-Effect Transistor Biosensors for Continuous and Multiplexed Neurotransmitter Detection**

*Bo Xiao^#^, Tingxian Li^#^, Xianmao Cao, Yang Zhang, Jianping He, Mengmeng Xiao^*^, Zhiyong Zhang^*^*

Bo Xiao, Jianping He, Mengmeng Xiao, Zhiyong Zhang

Hunan Institute of Advanced Sensing and Information Technology

Xiangtan University

Hunan, 411105, China

E-mail: mmxiao@pku.edu.cn and zyzhang@pku.edu.cn

Tingxian Li, Mengmeng Xiao, Zhiyong Zhang

Key Laboratory for the Physics and Chemistry of Nanodevices and Center for Carbon-based Electronics, Department of Electronics

Peking University,

Beijing 100871, China

Xianmao Cao, Yang Zhang

School of Integrated Circuits

Beijing University of Posts and Telecommunications

Beijing 100876, China

^#^These authors contribute equally

Corresponding author: mmxiao@pku.edu.cn and zyzhang@pku.edu.cn


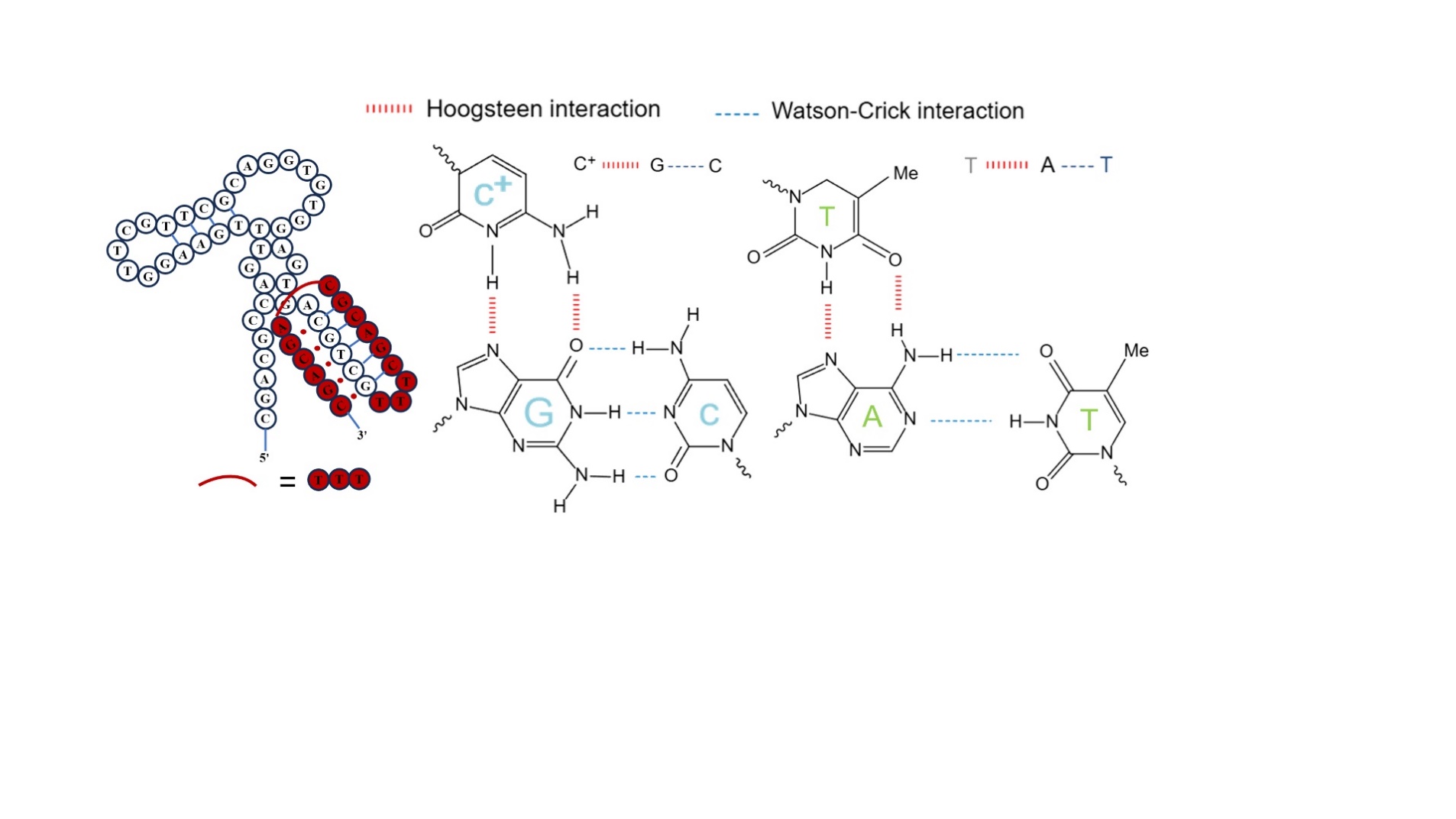


**Figure S1.** Schematic representation of a dopamine aptamer with a pH-sensitive fragment. Reversible double to triple chain conversion by formation of intramolecular Hoogsteen interactions in an acidic environment.


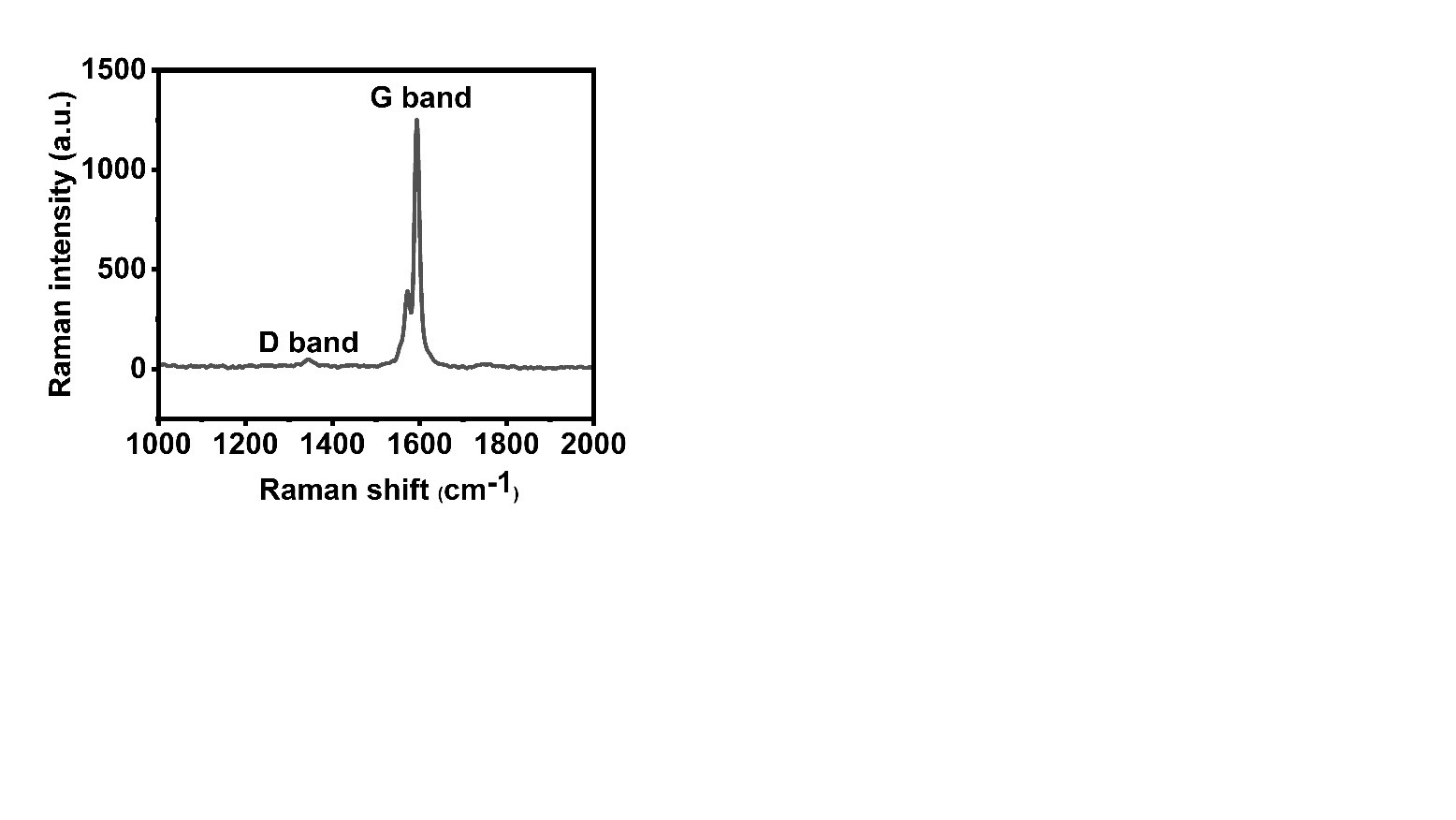


Figure S2. Raman characterization of carbon nanotubes.


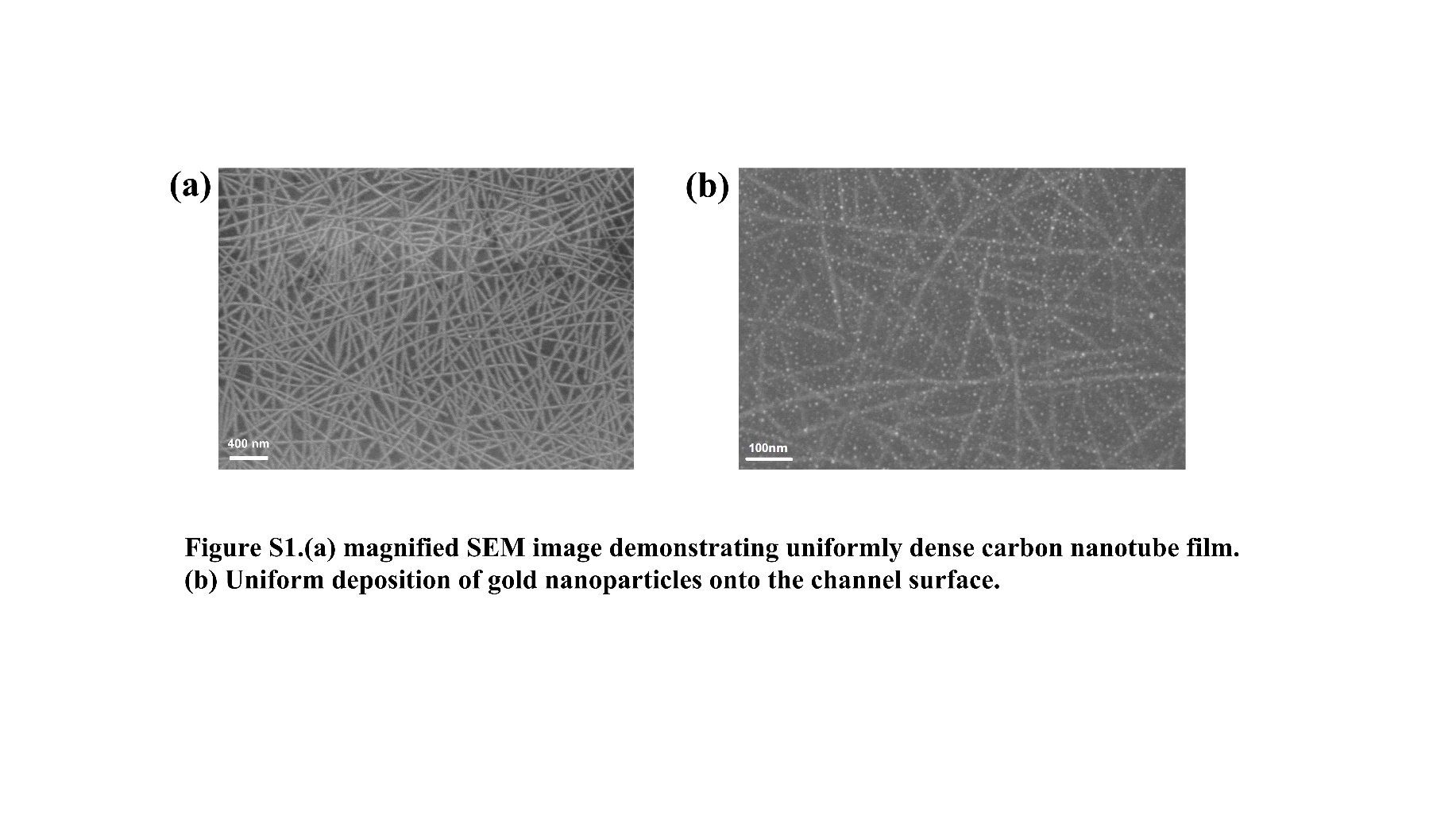


**Figure S3**. SEM characterization of CNTs. (a) Magnified SEM image demonstrating uniformly dense carbon nanotube film. (b) Uniform deposition of gold nanoparticles onto the channel surface.


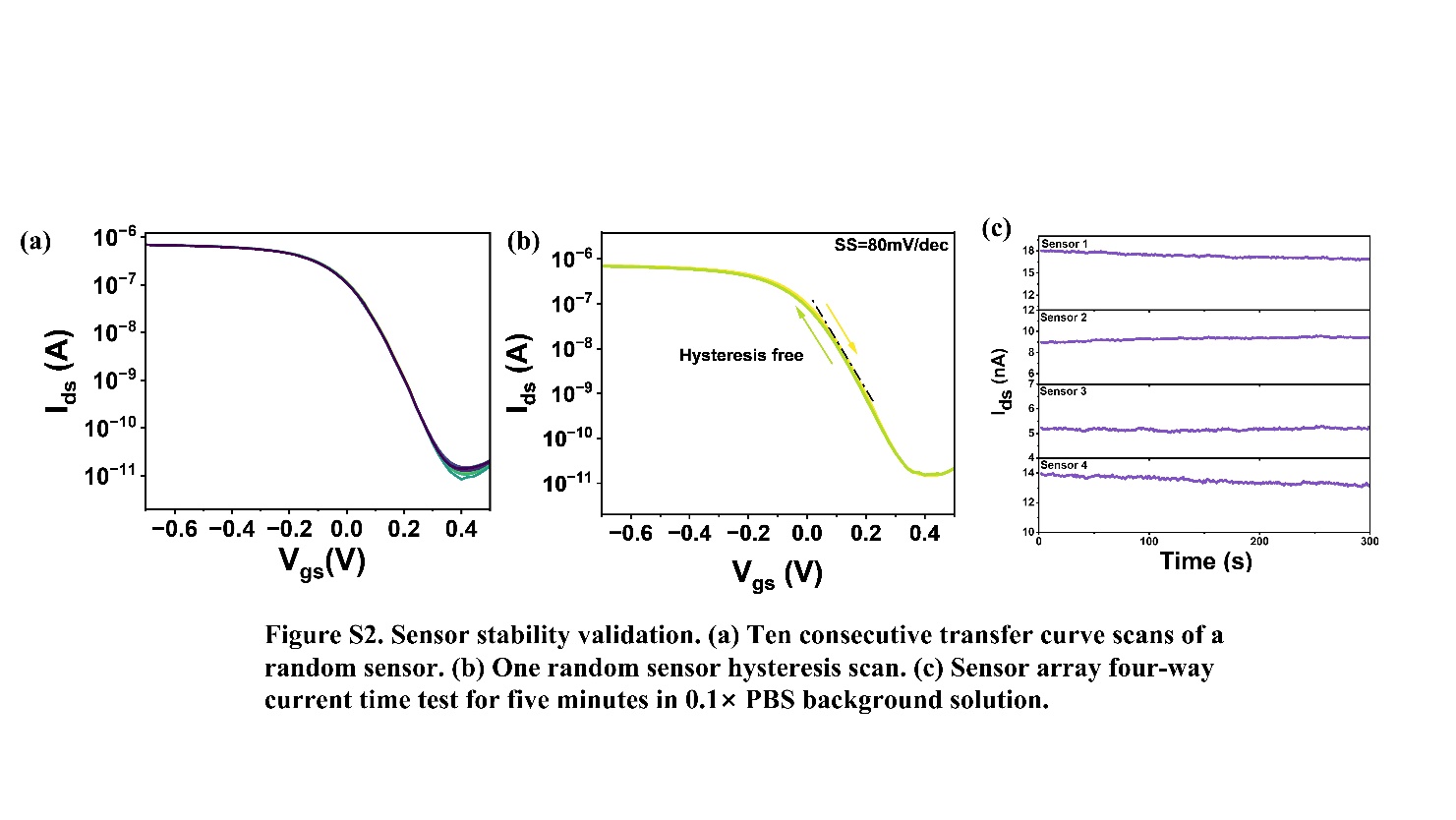


**Figure S4.** Sensor stability validation. (a) Ten consecutive transfer curve scans of a random sensor. (b) One random sensor hysteresis scan. (c) Sensor array four-way current time test for five minutes in 0.1× PBS background solution.


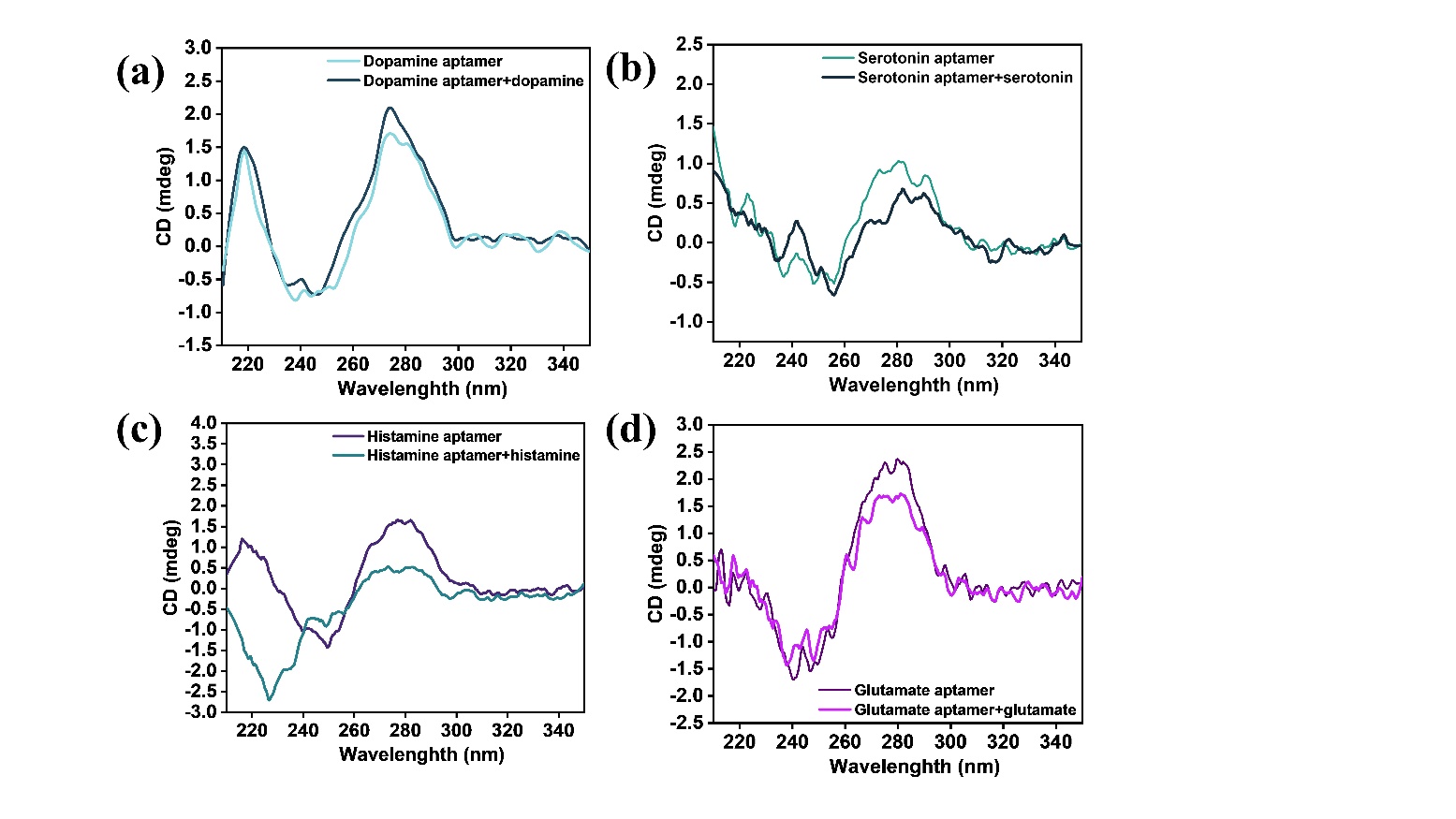


**Figure S5.** Changes in aptamer secondary structures upon adaptive binding to small-molecule targets. (a) Circular dichroism spectroscopy of dopamine aptamers after target capture. (b) Circular dichroism spectroscopy of serotonin aptamers after target capture. (c) Circular dichroism spectroscopy of histamine aptamers after target capture. (d) Circular dichroism spectroscopy of glutamate aptamers after target capture.


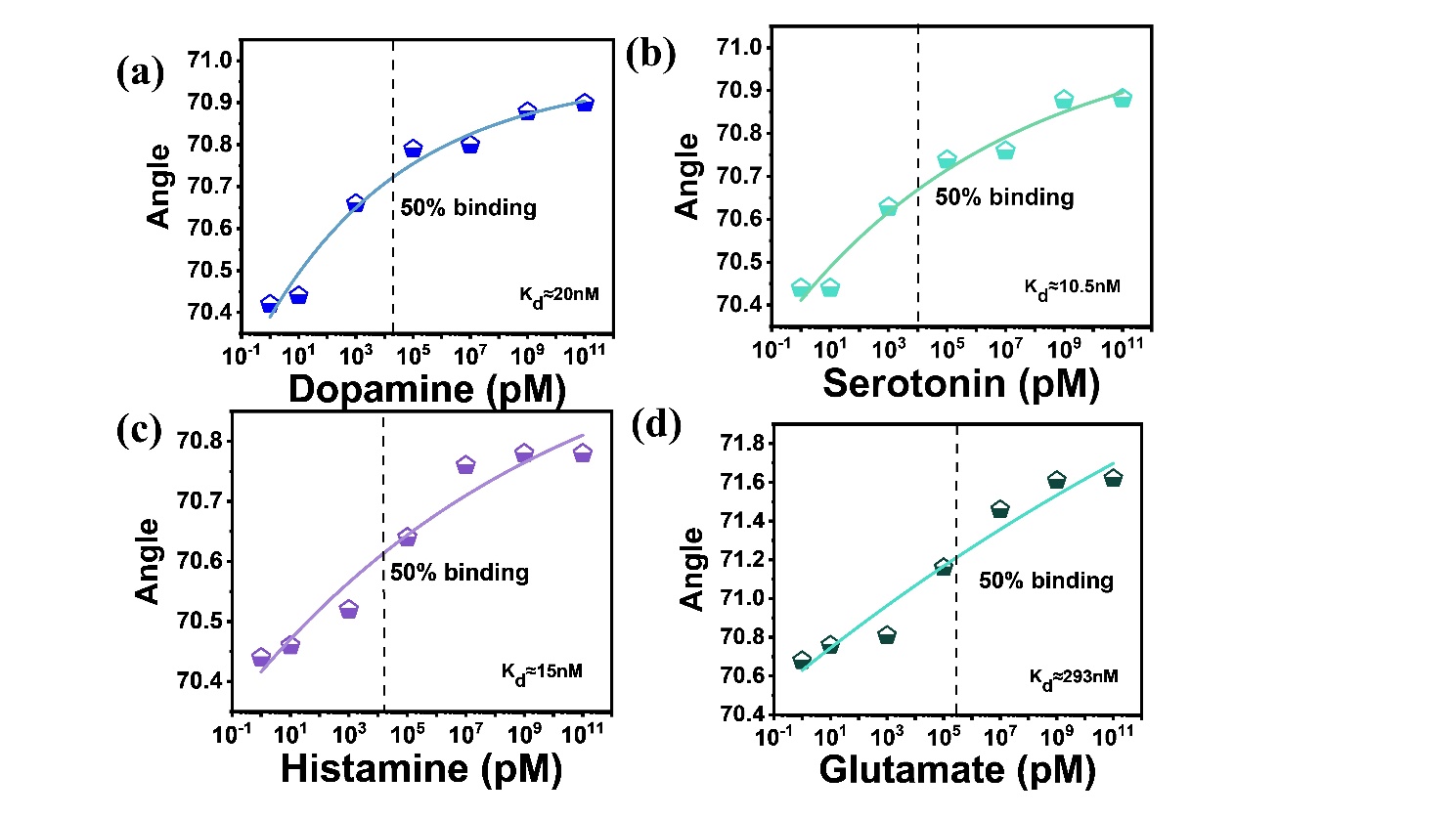


**Figure S6.** Determination of aptamer binding constants (K_d_). (a) Dopamine aptamer affinity calibration curve. (b) Serotonin aptamer affinity calibration curve. (c) Histamine aptamer affinity calibration curve. (d) Glutamate aptamer affinity calibration curve.


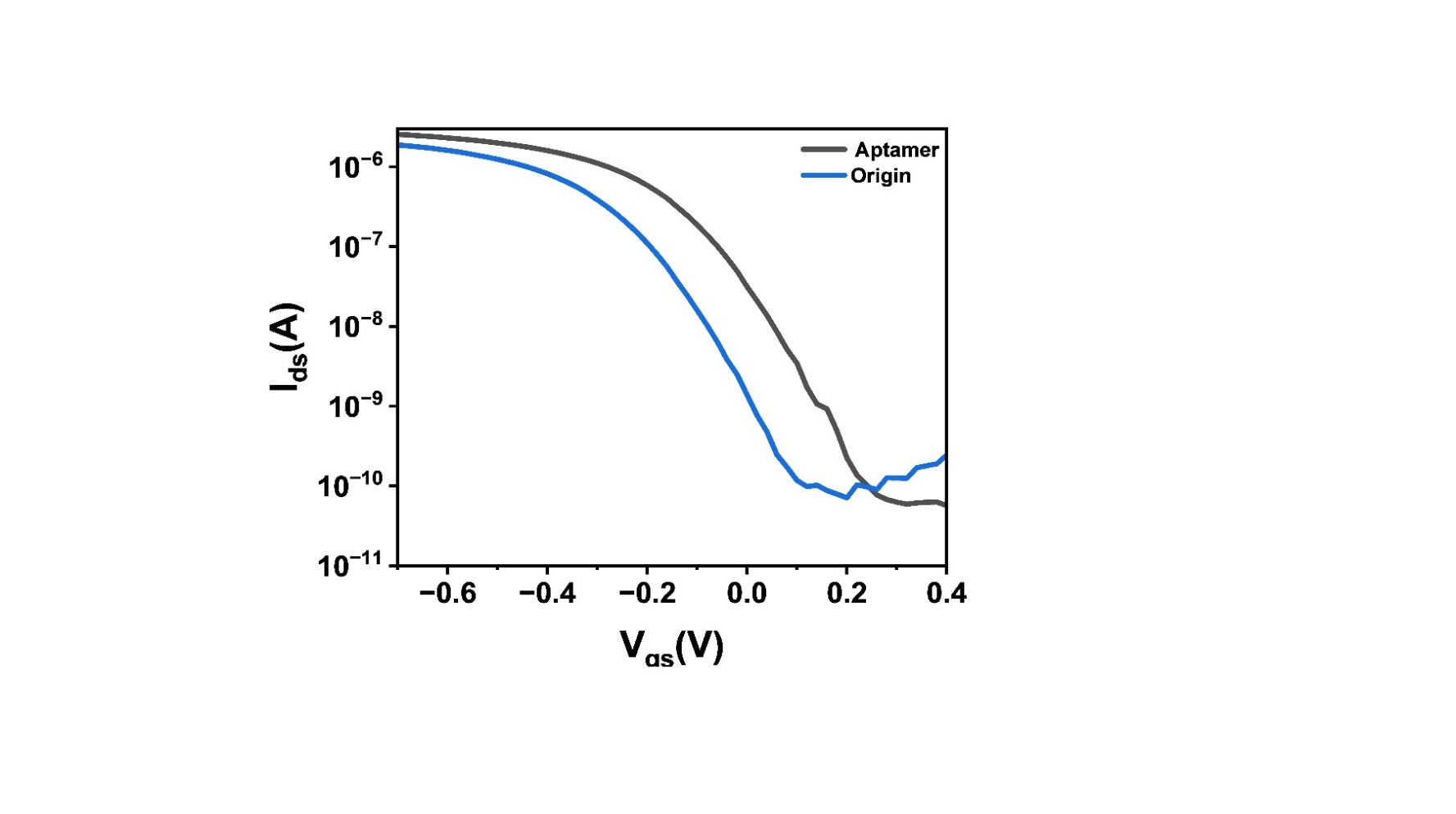


**Figure S7.** Change in transfer curve before and after sensor modification.


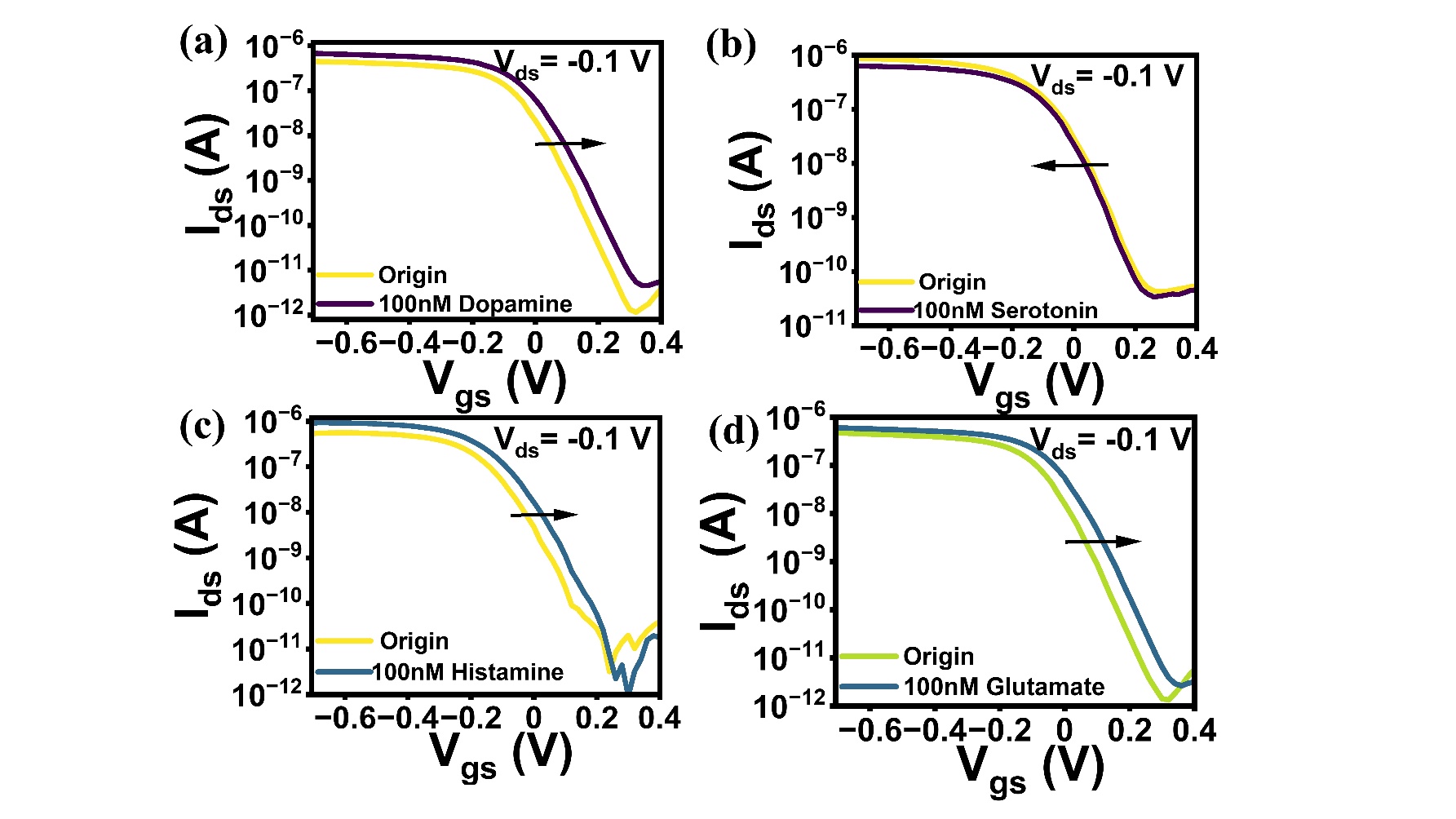


**Figure S8.** Sensor response to a single neurotransmitter. (a) Dopamine sensor response to dopamine; (b) Serotonin sensor response to serotonin; (c) Histamine sensor response to histamine; (d) Glutamate sensor response to glutamate.


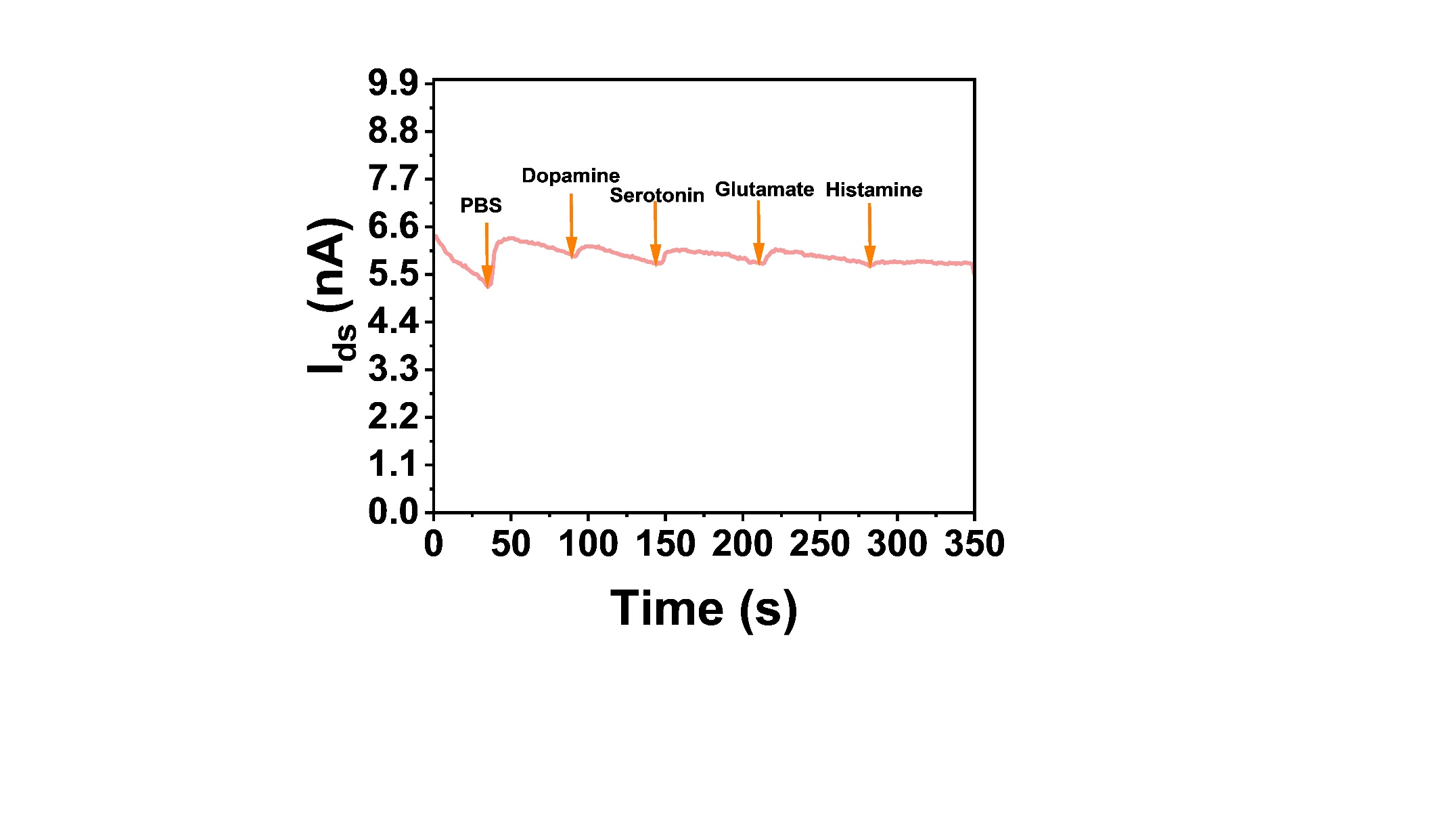


**Figure S9.** Sensors without modified aptamers responded to 0.1× PBS, 10 nM dopamine, 10 nM serotonin, 10 nM glutamate, and 10 nM histamine.


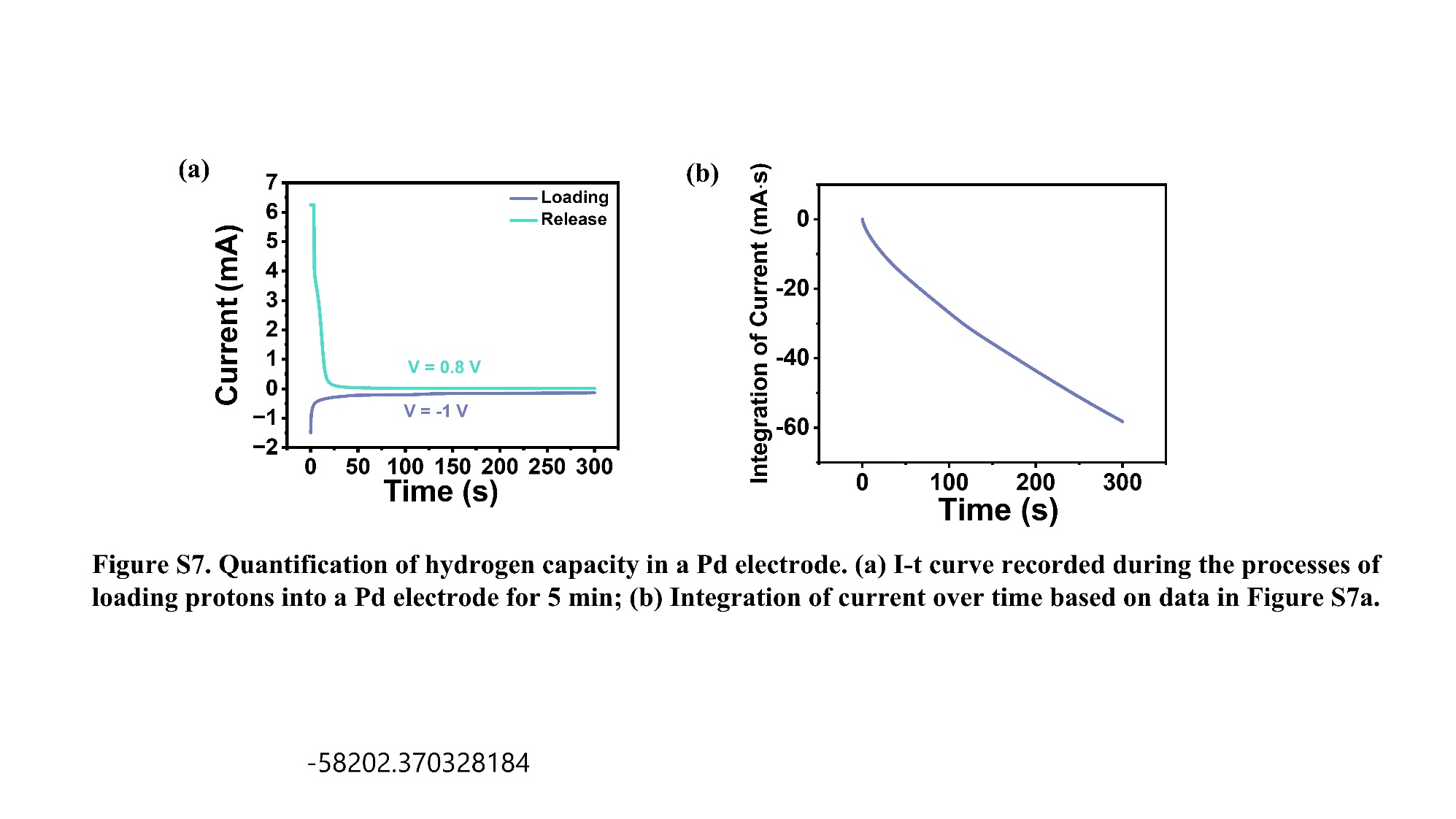


**Figure S10.** Quantification of hydrogen capacity in a Pd electrode. (a) I-t curve recorded during the processes of loading protons into a Pd electrode for 5 min; (b) Integration of current over time based on data in Figure S10a.

**Supplementary Note S1**

Calculation of H^+^ Capacity of a Pd electrode (diameter: 2 mm)

During the H^+^ loading process, the following reaction occurs:

$H^{+}+e^{-}\to H$ (1)

The total number of electrons (*n*) transferred during the process can be calculated using the equation:

$\int I dt = n \times e$ (2)

Where *I* is the measured current, *t* is the duration of electrochemical actuation, and *e* is the elementary charge (1.6×10^−19^ C).

Based on the data in Figure S7 (*t* ≈300s), the integral ∫ *I dt* is 0.058 A⋅s. Thus, the value of *n* is calculated as:

$n =\frac{0.058 A \cdot s}{1.6 \times{10}^{-19} C} = 3.625 \times{10}^{17}$ (3)

Dividing *n* by Avogadro's constant (6.022×10^23^ mol^−1^) yields the H⁺ capacity of the Pd electrode:

$H⁺ capacity=\frac{3.625\times{10}^{17}}{6.022\times{10}^{23}}=6.02\times{10}^{-7}mol$ (4)


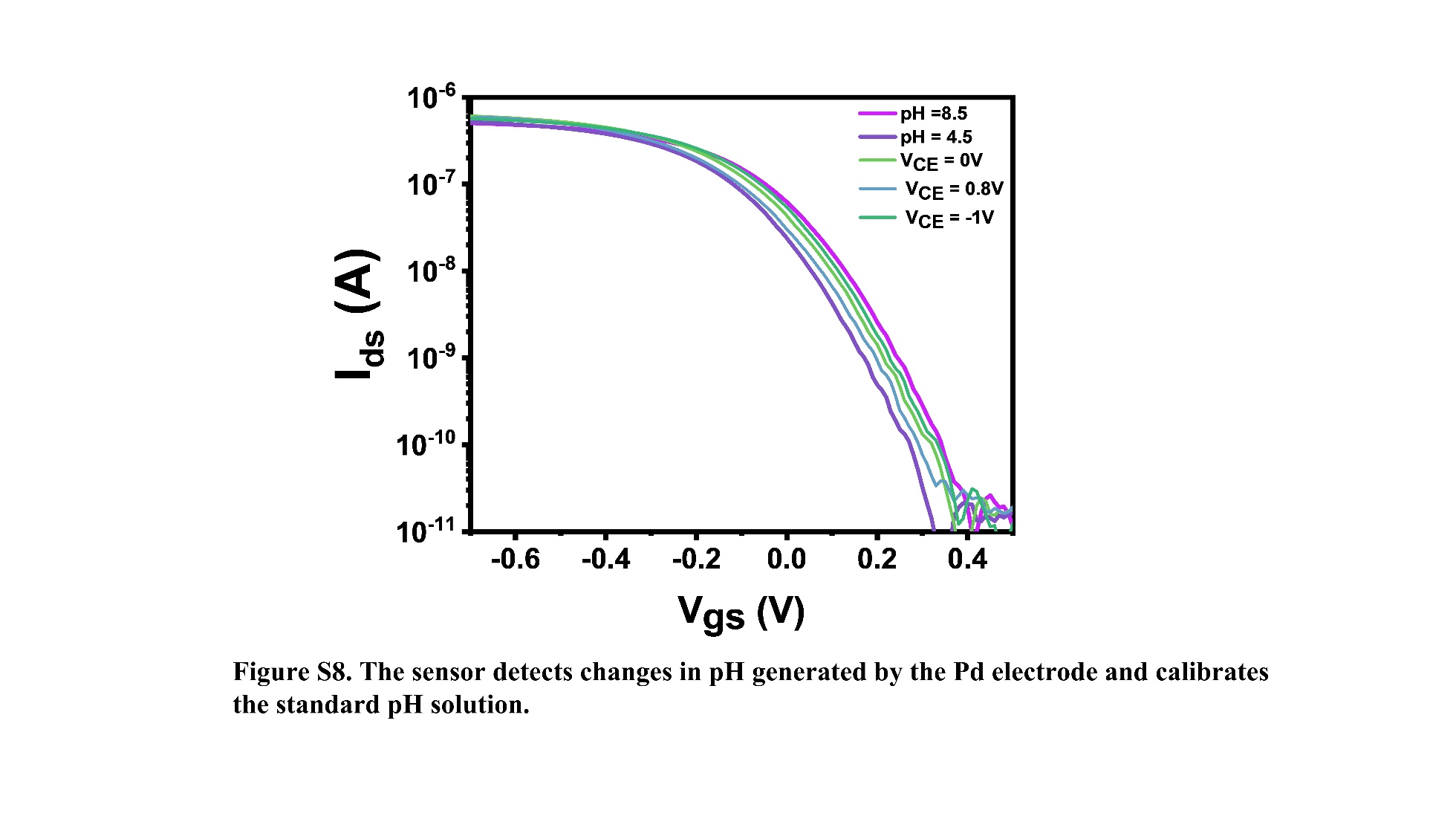


**Figure S11.** The sensor detects changes in pH generated by the Pd electrode and calibrates the standard pH solution.

**
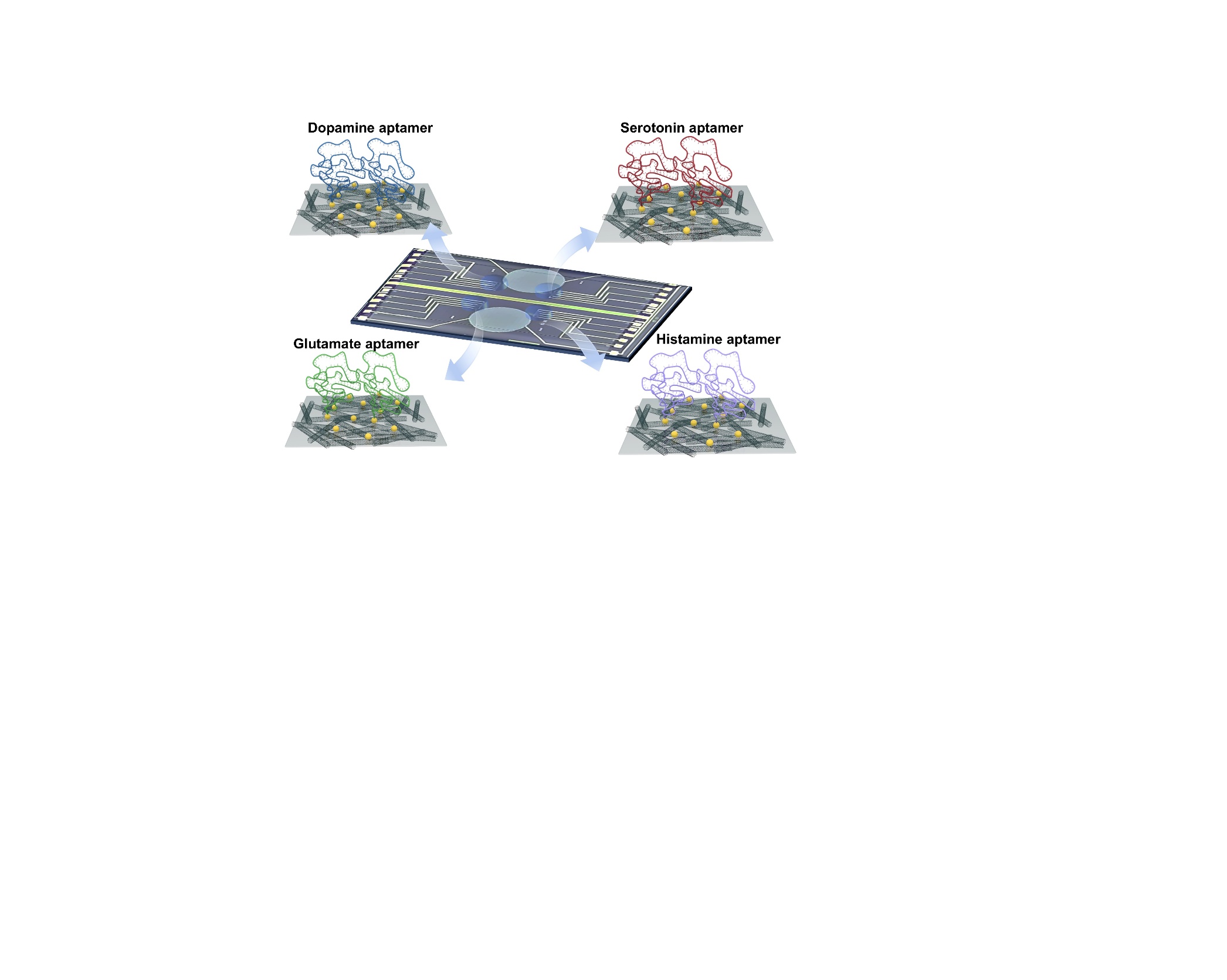
**

**Figure S12.** Schematic of subregional modifications of four neurotransmitter aptamers. The sensor array is divided into four distinct regions, with each region modified with a unique aptamer specific to a particular neurotransmitter. This subregional modification enables targeted detection of multiple analytes.


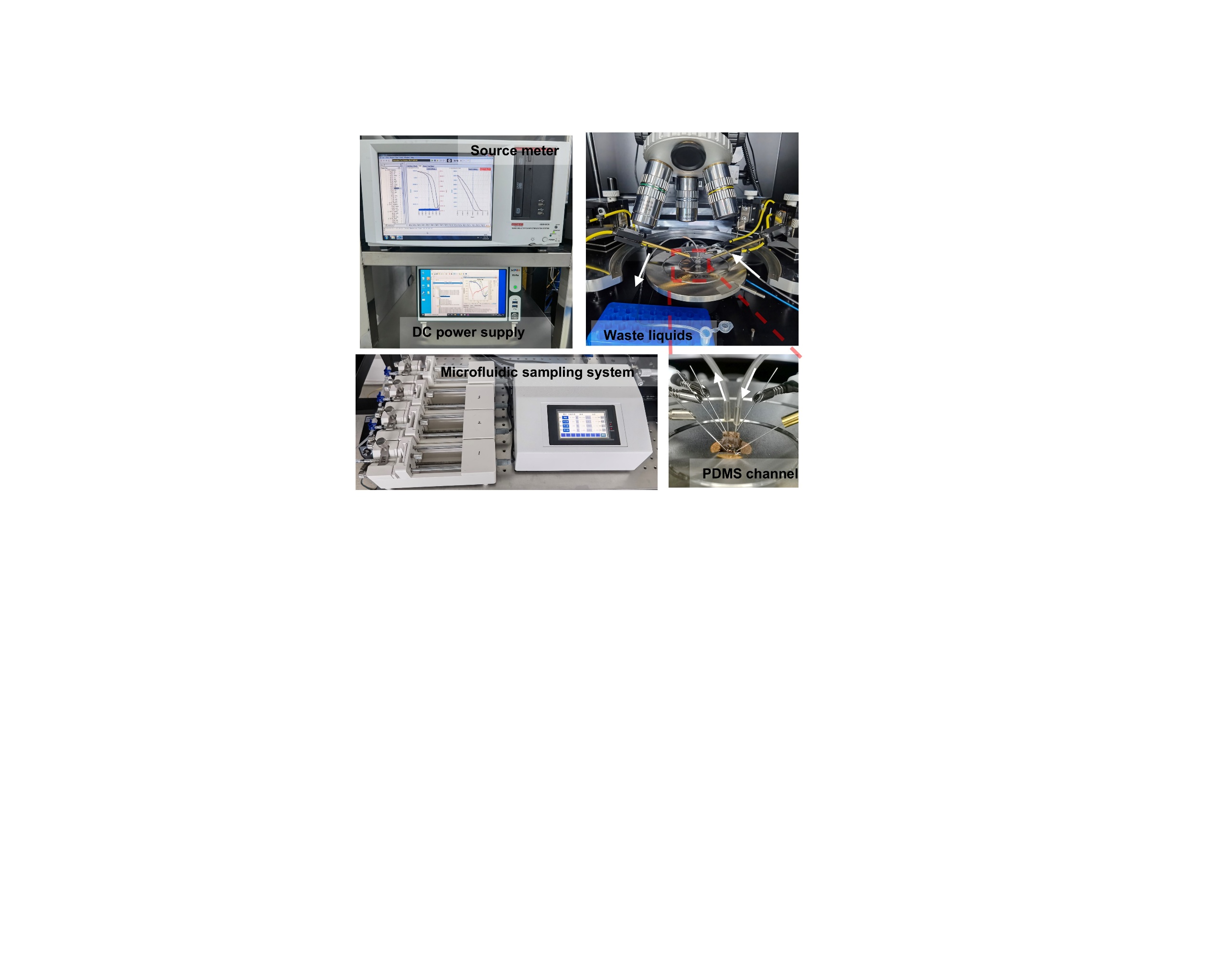


**Figure S13.** Schematic of multi-target assay for simultaneous detection of four targets Using microchannels and an additional voltage control module. A PDMS microchannel is fixed atop the sensor array, and sample injection is performed using an injection pump. All waste liquid is directed into a single waste reservoir via a unified outlet.


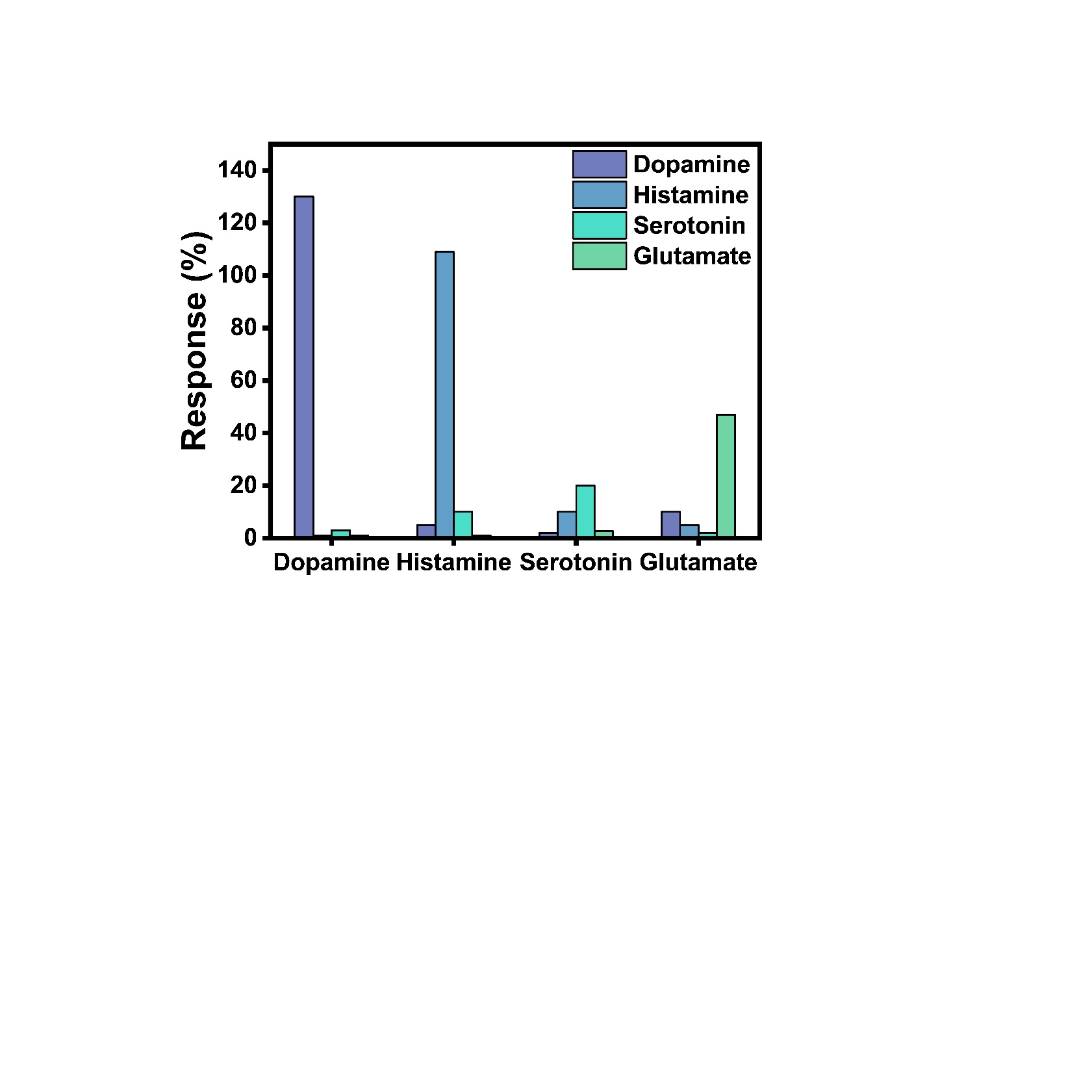


**Figure S14.** Extraction of sensor responses during simultaneous detection of different analytes. Upon stabilization of each sample, the responses of the four sensors are recorded.


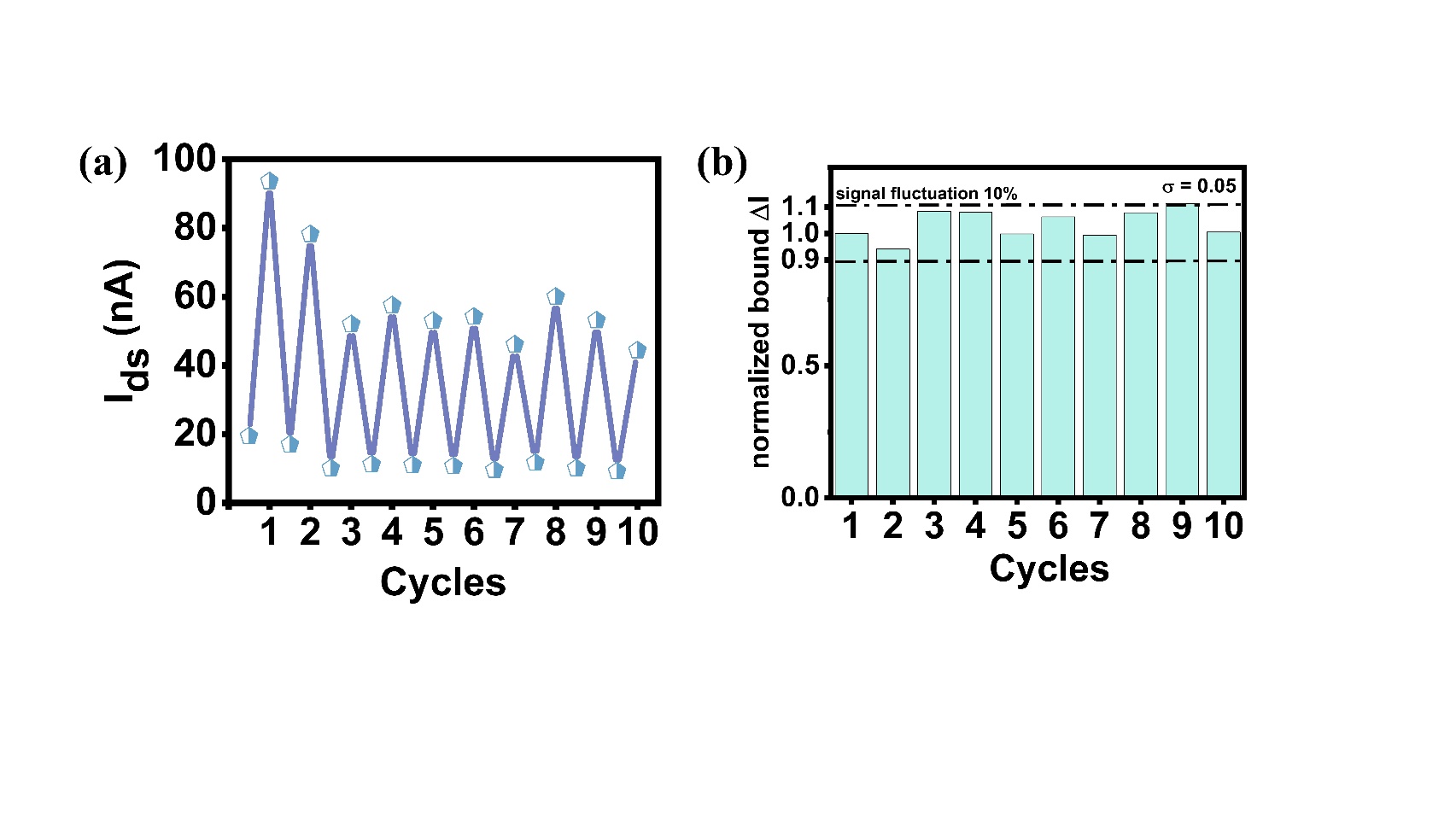


**Figure S15.** Recoverability test of the sensor to pH changes. (a)Repeatable test for ten I_ds_ changes; (b) The effect of repetitive regenerations on the sensor's functionality. The ten-cycle response fluctuates over a 10% range.

| **Aptamer** | **Sequences (5′ → 3′)** |
| --- | --- |
| **Dopamine** | HS-C6-CGACGCCAGTTTGAAGGTTC  GTTCGCAGGTGTGGAGTGACGTCG |
| **Serotonin** | HS-C6-CGACTGGTAGGCAGATAGGG  GAAGCTGATTCGATGCGTGGGTCG |
| **Histamine** | HS-C6-AGCTCCAGAAGATAAATTACAGGGAACGT  GTTGGTTGCGGTTCTTCCGATCTGCTGTGTTCTC  TATCTGTGCCATGCAACTAGGATACTATGACCCCGG |
| **Glutamate** | HS-C6-GCATCAGTCCACTCGTG  AGGTCGACTGATGAGGCTCGAT |
| **Dopamine**  **pH-sensitive** | HS-C6-CGACGCCAGTTTGAAGGTTCGTTCGCAG  GTGTGGAGTGACGTCGTTTCGACGCTTTAGCAGC |
| **Glutamate**  **pH-sensitive** | HS-C6-GCATCAGTCCACTCGTGAGGTCGA  CTGATGAGGCTCGATTTTATCGATTTAGCTA |
| **Serotonin**  **pH-sensitive** | HS-C6-CGACTGGTAGGCAGATAGGGGAAGCT  GATTCGATGCGTGGGTCGTTTCGACCTTTCCAGC |
| **Histamine**  **pH-sensitive** | HS-C6-AGCTCCAGAAGATAAATTACAGGGA  ACGTGTTGGTTGCGGTTCTTCCGATCTGCTGTGTTCTCTATCTGTGCCATGCAACTAGGATACTATGACCCCGGTTTCCGGGGTTTTTGGGGCC |

**Table S1.** The sequences of aptamer used in this work. The pH-sensitive dopamine aptamer forms a triple-stranded sequence of CGTCG, and the pH-sensitive glutamate aptamer forms a triple-stranded sequence of TCGAT，the pH-sensitive Serotonin aptamer forms a triple-stranded sequence of GGTCG, the pH-sensitive Histamine aptamer forms a triple-stranded sequence of ACCCCGG.

References

[1] Z. Hu, Y. Li, G. Figueroa-Miranda, S. Musall, H. Li, M. A. Martínez-Roque, Q. Hu, L. Feng, D. Mayer, A. Offenhäusser, Aptamer based biosensor platforms for neurotransmitters analysis，*TrAC Trends in Analytical Chemistry* **2023**, *162*, 117021.

[2] N. Nakatsuka, K.-A. Yang, J. M. Abendroth, K. M. Cheung, X. Xu, H. Yang, C. Zhao, B. Zhu, Y. S. Rim, Y. Yang, P. S. Weiss, M. N. Stojanović, A. M. Andrews, Aptamer–field-effect transistors overcome Debye length limitations for small-molecule sensing *Science* **2018**, *362*, 319.
